# A description of the clinical signs and lesions of African swine fever, and its differential diagnoses in pigs slaughtered at selected abattoirs in central Uganda

**DOI:** 10.3389/fvets.2025.1568095

**Published:** 2025-05-29

**Authors:** John E. Ekakoro, Aisha Nassali, Cole Hauser, Krista Ochoa, Dickson Ndoboli, Rodney Okwasiimire, Edrine B. Kayaga, Eddie M. Wampande, Karyn A. Havas

**Affiliations:** ^1^Department of Public and Ecosystem Health, College of Veterinary Medicine, Cornell University, Ithaca, NY, United States; ^2^Department of Veterinary Biomedical Sciences, Shreiber School of Veterinary Medicine, Rowan University, Mullica Hill, NJ, United States; ^3^Central Diagnostic Laboratory, College of Veterinary Medicine, Animal Resources and Biosecurity, Makerere University, Kampala, Uganda

**Keywords:** African swine fever, clinical signs and lesions, differential diagnoses, pigs, Uganda

## Abstract

**Introduction:**

African swine fever (ASF) is a contagious and hemorrhagic viral disease of pigs that may present as a per-acute, sub-acute or chronic disease. Prior to this study, the clinical and pathologic presentation of ASF in pigs slaughtered in Uganda had not been characterized, and studies varied in their findings regarding differential diagnoses. The objectives of this study were to: (1) describe the clinical and pathologic presentation of ASF in pigs sampled from abattoirs in the Kampala metropolitan area over the course of one year, and (2) determine the prevalence of swine influenza A viruses (S-IAV), porcine reproductive and respiratory syndrome virus (PRRSV), classical swine fever virus (CSFV), and *Salmonella* spp. in these pigs.

**Methods:**

Clinical and pathological data and samples were collected from pig abattoirs located in the Kampala metropolitan area from May 2021 through June 2022. Confirmatory diagnostic testing for African swine fever virus (ASFV) was performed using the real-time PCR (qPCR) assay. Diagnostic testing for ASFV differential diagnoses were performed using serologic and molecular techniques.

**Results:**

Severe fever was found in 3.3% (26/794) of all pigs that were ASFV positive by any of the sample types tested. Of 196 blood positive pigs, 26% (51) had widespread splenic hemorrhages compared to 15.2% (67/442) of the pigs positive based on testing of lymph nodes, 15.5% (72/464) of pigs positive based on tonsil samples, and 15.8% (61/385) of pigs with positive spleen samples. The median gross pathologic lesion score for all pigs that tested positive for any sample type was six out of 33 [interquartile range (IQR): 4, 9]. Overall, 89.3% of the pig samples (1,188/1,330) were seropositive for S-IAV, and 0.8% (11/1,329) were seropositive for PRRSV. As for *Salmonella* spp., 4.4% (40/903) were qPCR positive, and all samples tested for CSFV nucleic acid were negative.

**Conclusion:**

ASF in pigs slaughtered in central Uganda presents with clinical signs and lesions that vary; they present as healthy pigs or pigs with subacute or acute disease. However, surveillance programs in Uganda will require confirmatory laboratory diagnosis due to the occurrence of pathogens that cause similar clinical signs and lesions.

## 1 Introduction

African swine fever (ASF) is a highly contagious and hemorrhagic disease of pigs caused by the African swine fever virus (ASFV) belonging to the family *Asfarviridae* (1, 2). African swine fever was first described in Kenya in 1921 (3) and is known to be endemic in Uganda (4). The clinical presentation and pathological lesions of ASF may vary based on the virulence of the infecting virus, the route and dose of infection, and the host characteristics (5). Clinically, ASF may present as per-acute, acute, sub-acute, or chronic disease (6). Highly virulent strains of ASFV cause per-acute and acute forms of the disease, acute and subacute disease is caused by moderately virulent strains, and the chronic form of ASF is associated with moderate to low virulence isolates (5).

The per-acute form of ASFV infection is characterized by a rapid clinical course, coupled with high fever, anorexia, lethargy, and occasionally sudden death with no clinical signs, and no gross lesions observed during post-mortem examination (6, 7). The acute form presents with clinical signs and pathologic lesions such as, loss of appetite, high fever, pulmonary edema, extensive necrosis and hemorrhage of lymphoid tissue, and hemorrhages in the skin (6). Hemorrhagic splenomegaly is the most characteristic pathologic lesion (6). The subacute form of ASF resembles the acute form although with less severe clinical signs and more intense hemorrhage and edema (5). In animals with subacute disease, hydropericardium, and ascites are observed post-mortem and multifocal edema of the wall of the gallbladder or the perirenal fat is also observed (6). A review of the key ASF gross and microscopic pathologic features showed that chronic ASF is characterized by multifocal skin necrosis, arthritis, growth retardation, loss of weight, respiratory distress, and abortion (6). This clinical form has been associated with naturally occurring ASFV isolates (8, 9) and low virulence isolates that are believed to have evolved from ASFV isolates employed in early vaccine trials carried out in the Iberian Peninsula in the 1960s (6, 10).

The clinical signs and lesions of ASF are similar to those observed with other pig diseases such as classical swine fever (CSF), septicemic salmonellosis, swine influenza, porcine reproductive and respiratory syndrome (PRRS) (5, 11). Clinically, both ASF and CSF present with high fever, inappetence, incoordination, erythema (reddening of the skin), and disseminated hemorrhages predominantly in the lymph nodes (5, 11, 12). Just like ASF, septicemic salmonellosis may present with signs of febrile disease, e.g., fever and reduced feed intake, lymph node and spleen enlargement, and skin cyanosis (5, 11). The clinical signs and pathologic lesions observed in both ASF and swine influenza include fever, depression, reduced feed intake, coughing, nasal and ocular discharges, conjunctivitis, difficulty in breathing, lesions in the respiratory system such as pulmonary edema, airway exudates, lack of lung collapse (7, 11–14). Clinically, both ASF and PRRS may present with signs of respiratory disease (11, 15) and skin cyanosis, swollen or marbled lymph nodes, petechial hemorrages in the kidneys are observed in both diseases (5). In light of these similarities, clinical diagnosis of ASF based on clinical signs and lesions alone is difficult and unreliable. Thus, confirmatory laboratory testing is required for a definitive diagnosis of ASFV (7). Prior to this study, the clinical and pathologic presentation of ASF in pigs slaughtered in the Kampala metropolitan area of Uganda had not been characterized. Additionally, in Uganda, little was known about the occurrence of pathogens such as swine influenza A viruses (S-IAV), porcine reproductive and respiratory syndrome virus (PRRSV), classical swine fever virus (CSFV), and *Salmonella* spp., all of which cause clinical disease resembling ASF. The objectives of this study were to: (1) describe the clinical & pathologic presentation of ASF in pigs sampled from abattoirs in the Kampala metropolitan area over the course of one year, and (2) determine the prevalence of S-IAV, PRRSV, CSFV, and *Salmonella* spp., in pigs slaughtered in central Uganda.

## 2 Results

### 2.1 Characteristics of the pigs

Data and samples were collected from 1,334 sampled pigs that originated from 44 out of 146 (30.1%) districts of Uganda. Most of the ASFV positive pigs were sampled at the Wambizi (33%) and Lusanja (31.9%) abattoirs, 54.4% of the 794 positive pigs were exotic breed type, and most of the pigs (43.8%) came from smallholder farmers raising 1–3 pigs. Table 1 summarizes the characteristics of the ASF positive pigs.

**Table 1:** Summary of the characteristics of ASFV qPCR positive pigs sampled from May 2021 through June 2022 from six abattoirs located in the Kampala metropolitan area of Uganda.

Characteristics of pigs	Number positive (%)	Number negative (%)	Total
Total number of pigs that tested positive	794 (59.5)	540 (40.5)	1,334
Abattoir of slaughter			
Lusanja	253 (57.1)	190 (42.9)	443
Wambizi	263 (66.4)	133 (33.6)	396
Buwate	49 (47.6)	54 (52.4)	103
Katabi	118 (60.5)	77 (39.5)	195
Budo	89 (65.9)	46 (34.1)	135
Kyetume	22 (35.5)	40 (64.5)	62
Pig breed type			
Local	132 (65.7)	69 (34.3)	201
Exotic	432 (56.8)	328 (43.2)	760
Mixed	219 (62.2)	133 (37.8)	352
Unknown	11 (52.4)	10 (47.6)	21
Sex of pig			
Female	433 (59.4)	296 (40.6)	729
Male	356 (59.5)	242 (40.5)	598
Unknown	5 (71.4)	2 (28.6)	7
Pre-slaughter length of stay at abattoir			
Arrived on day of slaughter	50 (60.2)	33 (39.8)	83
Arrived the previous day	329 (57.9)	239 (42.1)	568
Spent 2–5 days at abattoir	236 (51.4)	223 (48.6)	459
Spent longer than a week at abattoir	13 (61.9)	8 (38.1)	21
Unknown duration	166 (81.8)	37 (18.2)	203
Where sourced			
From a smallholder farmer with herd size 1–3 pigs	348 (63.3)	202 (36.7)	550
From a medium-scale farmer (4–11 pigs)	144 (58.5)	102 (41.5)	246
From a large-scale farmer (>11 pigs)	256 (55.5)	205 (44.5)	461
From a livestock market	13 (61.9)	8 (38.1)	21
Unknown	33 (58.9)	23 (41.1)	56

### 2.2 Clinical signs observed antemortem

**Figure 1:**
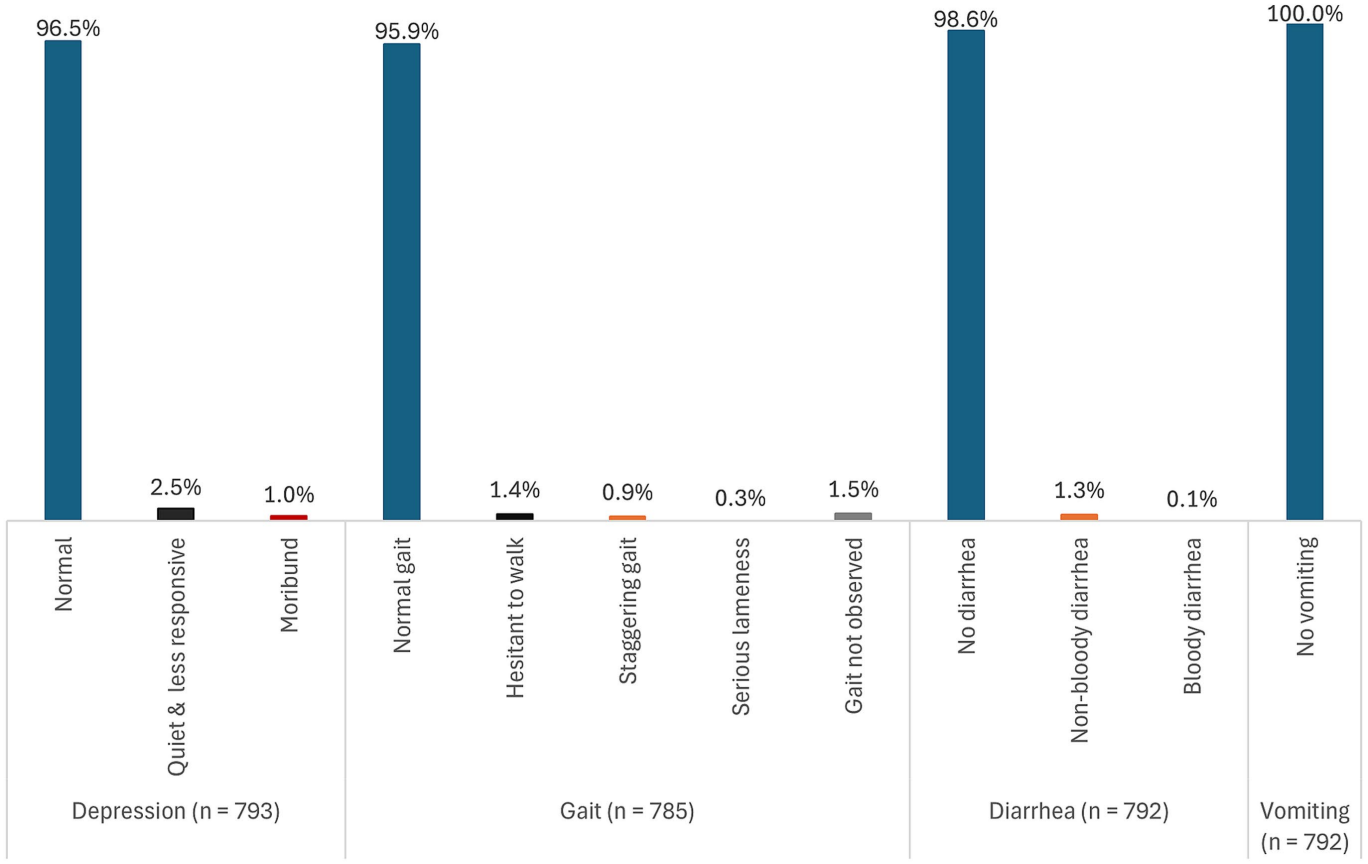
Distribution of clinical signs (depression, gait, diarrhea, and vomiting) in pigs that were ASFV qPCR positive by any of the sample types tested.

**Figure 2:**
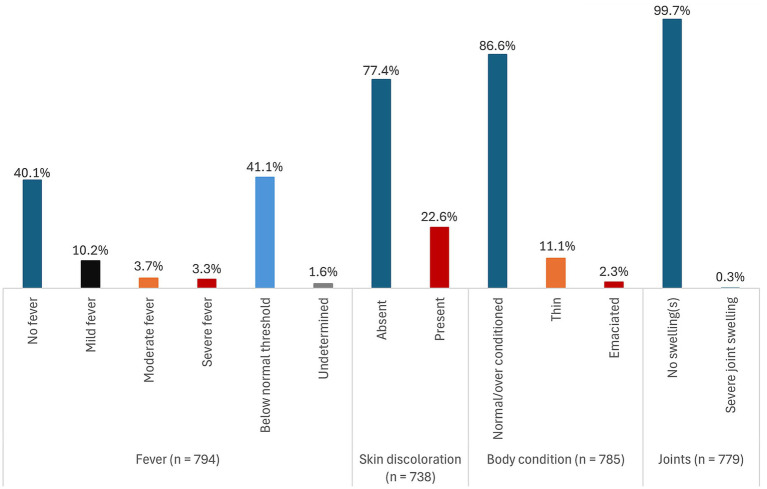
Distribution of clinical signs (fever, skin discoloration, body condition, and joint characteristics) in pigs that were ASFV qPCR positive by any of the sample types tested.

**Figure 3:**
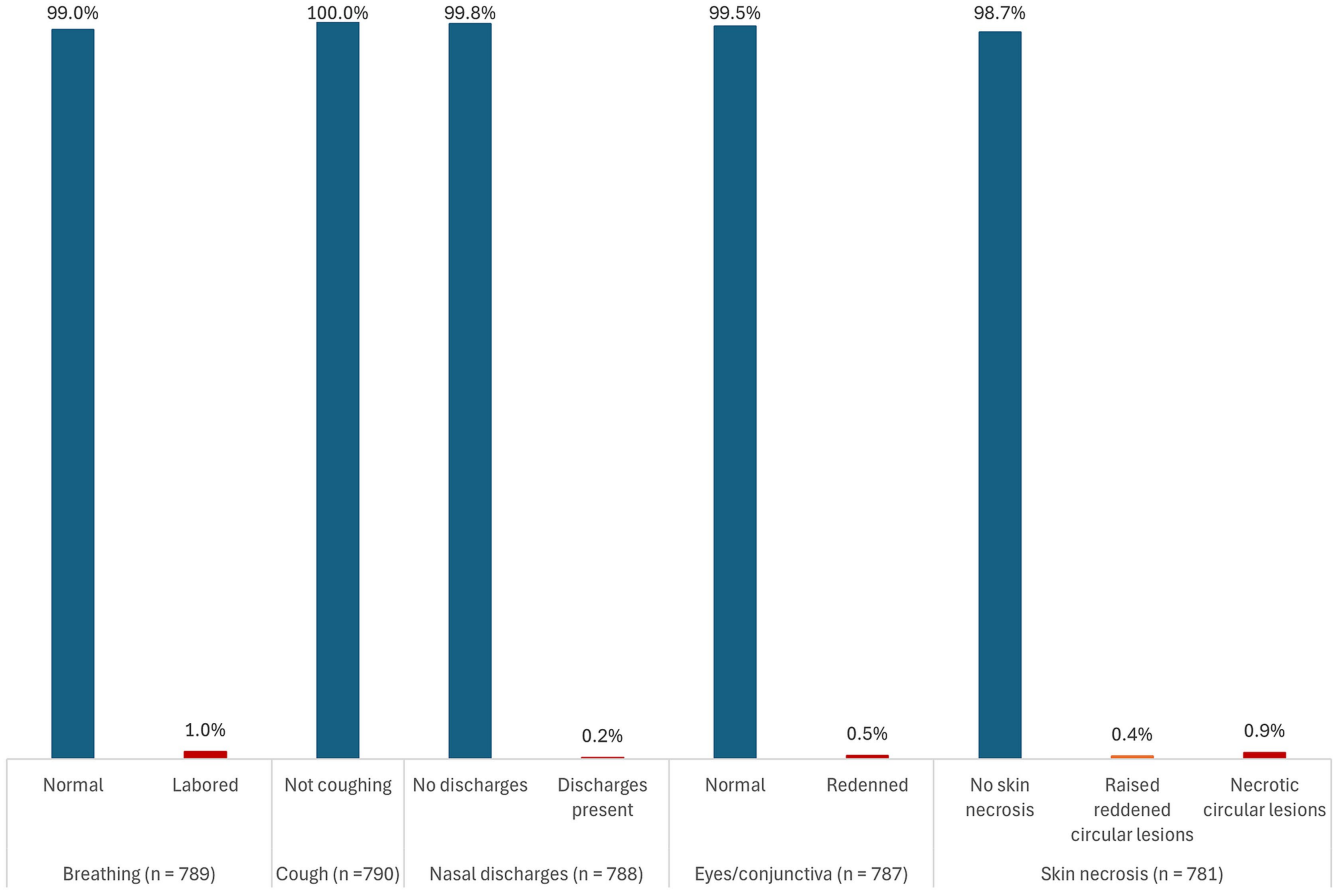
Distribution of clinical signs (breathing, cough, nasal discharges, eyes/conjunctiva, and skin necrosis) in pigs that were ASFV qPCR positive by any of the sample types tested.

Of the 1,334 pigs sampled and tested for ASFV using real-time PCR (qPCR), 59.5% (794) tested positive for ASFV for any sample type (blood, tonsil, lymph node, or spleen) collected. Figures 1–3 show the distribution of clinical signs observed in pigs that were ASFV qPCR positive by any of the sample types tested following antemortem examination. The majority of ASFV positive pigs were lively with no signs of depression (>90% regardless of sample type), had well-coordinated movements (>90% regardless of sample type), and had a normal or over-conditioned body score (>80% regardless of sample type).

Rarely found clinical signs regardless of sample type included evidence of non-bloody diarrhea (<2%), conjunctivitis (<1%), labored breathing (<2%), skin necrosis (<4%), and severe fever (<5%). No pigs had signs of vomiting and coughing. Overall, 41.1% (326/794) pigs had rectal temperatures below the normal threshold. However, there was no evidence that subnormal temperatures were associated with severe illness.

There were very little clinical signs associated with chronic disease detected. Severe joint swellings and lameness were observed in 0.2% (1/441) of lymph node positive pigs and in 0.4% (2/464) of tonsil positive pigs. Joint swellings were absent in blood and spleen positive pigs. The most common clinical sign was reddening of the skin. It was observed in 22.6% (167/738) of all pigs that were ASFV positive by any of the sample types tested, 31.5% (58/184) of blood positive pigs, 22.5% (95/423) of lymph node positive pigs, 25.3% (110/435) of tonsil positive pigs, and in 26.7% (98/367) of the spleen positive pigs. A detailed summary of the distribution of the clinical signs stratified by each of the sample types tested is presented in Supplementary file 1 (Supplementary Table S1).

### 2.3 Gross pathologic lesions

Pathologic lesions typical of acute ASF were found and are summarized in Supplementary file 1 (Supplementary Table S2). This included edema of the lungs with 64.1 to 73.2% of having lung edema based on the sample type used to detect ASFV. There were lesser amounts of hemorrhage in the lung with 34 to 36.1% showing some signs of hemorrhage depending on the sample type used to diagnose them. Hydropericardium was found less commonly. It was seen most often in pigs diagnosed using spleen samples (21.2%) and least often in pigs diagnosed using blood samples (16.6%). Marked splenomegaly was found in 15.5% (31/200) of blood positive pigs, 8.4% (38/451) of lymph node positive pigs, 9.3% (44/473) of tonsil positive pigs, and 10.7% (42/392) of spleen positive pigs. Of 196 blood positive pigs with data on spleen hemorrhages, 26% (51) had very dark red to almost black hemorrhages over the entire spleen compared to 15.2% (67/442) of the lymph node positive, 15.5% (72/464) of tonsil positive pigs, and 15.8% (61/385) of spleen positive pigs.

Across sample types, a large proportion of pigs had hemorrhagic lymphadenitis. Of the 197 blood positive pigs, 25.9% (51) had enlarged and diffusely hemorrhagic gastro-hepatic lymph nodes compared to 16.9% (75/443) of lymph node positive pigs, 16.9% (79/468) of tonsil positive pigs, and 19.5% (75/385) of spleen positive pigs. Hemorrhages found in other lymph node types are presented in Supplementary Table S2 in Supplementary file 1. Widespread petechial hemorrhages on the kidney surface were found in 22.9% (46/201) of blood positive pigs, 22.3% (100/448) of lymph node positive pigs, 20.5% (96/469) of tonsil positive pigs, and 25% (98/393) of spleen positive pigs.

Other hemorrhages were most commonly found on the intestinal serosa (6 to 7.5%) compared to low rates of detection of hemorrhages on the urinary bladder (≤1.7%), pericardium (≤1.1%), renal fat (≤1.8%) and gall bladder (≤1.1%). Necrosis of the tongue that is previously reported as lesions seen in chronic ASF was absent in all pigs evaluated. However, pathologic tonsil lesions previously described in chronic ASF were observed in a few pigs. Thirteen out of 196 (7%) blood positive pigs, 3.4% (15/444) of lymph node positive pigs, 3% (14/463) of tonsil positive pigs and 3.7% (14/384) of spleen positive pigs had reddened tonsil and/or tonsil with marked exudate.

Table 2 gives a summary of the median gross pathologic severity scores by sample type and overall. The median lesion score for all pigs that tested positive for any sample type was six out of 33 [interquartile range (IQR): 4, 9]. For specific sample types, the median lesion for blood was eight (IQR: 4, 12), six for both lymph nodes (IQR: 4, 9) and tonsil (IQR: 4, 10), and seven for spleen (IQR: 4, 10). All sample types had a score of four at the 25th percentile, and blood samples showed the greatest spread in its distribution of scores. The Kruskal–Wallis test showed a statistically significant difference in median scores (*p* = 0.025). Although no significant pairwise differences were found with the *post-hoc* test (*p*-value <0.05), differences between lymph nodes and blood (*p* = 0.059) and lymph nodes and spleen (*p* = 0.0752) were influential. Lymph nodes had a lower median and 75th percentile score than both blood and spleen.

**Table 2:** Scores for gross pathologic lesions for all ASFV positive pigs sampled from Kampala metropolitan area abattoirs and for positive pigs by sample type tested, May 2021 through June 2022.

Sample type tested by qPCR positive type	Number positive	Gross pathologic lesion score^a^			
		25th percentile	50th percentile (Median)	75th percentile	Range
Overall	794	4	6	9	0–23
Blood	201	4	8	12	0–23
Lymph nodes	453	4	6	9	0–23
Tonsil	474	4	6	10	0–23
Spleen	395	4	7	10	0–23

a Total possible lesion severity score was 33.

### 2.4 Differential diagnoses

Table 3 summarizes the proportions of all positive samples for ASFV differential diagnoses and the proportions of ASFV positive pigs that tested positive for ASFV differential diagnoses. Overall, 89.3% of the pig samples (1,188/1,330) were seropositive for S-IAV and 0.8% (11/1,329) were seropositive for PRRSV. In addition, 4.4% (40/903) were positive for *Salmonella* spp. using qPCR and all the 559 samples tested for CSFV nucleic acid using reverse transcriptase qPCR (rt-qPCR) were negative. A high proportion (89.9%) of ASFV positive pigs were exposed to S-IAV, 4.6% had *Salmonella* spp. nucleic acid detected, while just 0.9% were exposed to PRRSV. The median gross pathologic lesion score for the *Salmonella* spp. positive pigs was 3.5 (IQR: 0, 9). The 25th percentile, median and maximum (16) pathologic lesion scores were lower than for the ASFV positive pigs.

**Table 3:** Prevalence of ASFV differential diagnoses among pigs sampled from May 2021 through June 2022 from six abattoirs located in the Kampala metropolitan area of Uganda.

Pathogen	Number tested	Number positive	%	95% confidence interval
Proportions for all pig samples tested for ASFV differential diagnoses				
PRRSV^a^	1,329	11	0.8	0.4–1.5
S-IAV^a^	1,330	1,188	89.3	87.5–90.9
*Salmonella* spp.	903	40^b^	4.4	3.3–6.0
CSFV	559	0	0	0–0.8
Proportions of ASFV qPCR positive pigs that tested positive for ASFV differential diagnoses				
PRRSV^a^	794	7	0.9	0.4–1.8
S-IAV^a^	794	714	89.9	87.6–91.8
*Salmonella* spp.	568	26^b^	4.6	3.1–6.6
CSFV	339	0	0	0–1.3

a Indicates serologic testing was done and results are seroprevalence. All other diagnostic assays were real-time PCR assays that detected the pathogen of interest’s nucleic acid.

b 26/40 (65%) of the Salmonella positive pigs were positive for ASFV.

## 3 Discussion

This study was part of a larger project that evaluated African swine fever presentation and distribution in Uganda. To our knowledge this study is the first to describe the clinical signs and pathologic lesions of ASFV and the prevalence of its differential diagnoses in slaughtered pigs in Uganda. The findings described in this study are based on comprehensive data collected over a period of 13 months at six pig abattoirs that have a wide catchment area in the country. We found mild to moderate clinical signs in ASFV positive pigs and severe pathologic lesions in ASFV positive pigs slaughtered in the Kampala metropolitan area of Uganda. There were generally no signs of chronic ASFV lesions or signs. We also found pigs with no clinical signs. These findings show that the ASFV genotype and strains circulating in Uganda can cause subacute to acute disease in domestic pigs. As part of this project, we analyzed 31 samples by Whole Genome Sequencing, and they were all identified as genotype IX, indicating that ASFV genotype IX is currently circulating in Uganda (17). The clinical presentation reported in this present study could be associated with genotype IX. It is important to note that the historic ASFV genotype IX has been maintained as genotype 9 in a proposed new genotype classification (18). Pigs without clinical signs or with mild clinical signs may represent pigs that were exposed and sent for slaughter, potentially as part of outbreak sell-offs. Since these pigs were slaughtered and not followed over time, it is not clear if they were pre-clinical or if the disease was sub-clinical. Selling of pigs during outbreaks following outbreaks (panic sales) is a commonly reported management strategy used by farmers to reduce economic losses due to ASF (16, 19, 20). Pig abattoirs in Uganda could be used as surveillance sites for identifying ASF outbreaks and pathologic lesions seem more reliable for syndromic surveillance than clinical signs. However, it is important to note that some pigs that tested negative for ASFV had hemorrhages in the spleen, lymph nodes, kidneys, lungs as well as lung edema and tracheal exudates. This is indicative of the presence of ASFV differential diagnoses, hence confirmation will require laboratory diagnosis.

We found a very high level of exposure to S-IAV and a very low level of exposure to PRRSV among the slaughtered pigs. This high level of exposure to S-IAV and very low exposure to PRRSV may reflect immunologic responses to natural infections as the authors are not aware of any pig vaccination programs in Uganda against any of the pathogens tested. A previous Ugandan study conducted between 2010 and 2011 found a seroprevalence of 4.6% for S-IAV and a prevalence of 1.4% using reverse transcriptase polymerase chain reaction (21). The difference in seroprevalence found between the present study and that of Kirunda et al. (21) could be due to the difference in the type of ELISA test used or could be reflective of the temporal changes in the epidemiology of S-IAV given close to a 10-year difference between the study periods. The indirect S-IAV ELISA used in the present study had a sensitivity of 87 and 89% specificity (22). Another study conducted in Lira and Masaka districts of Uganda in 2013 found S-IAV seroprevalence of 8.5% in Lira and 2% in Masaka, and for PRRSV they found a 1.7% seroprevalence in Lira and 1.3% in Masaka (23). When compared to the Lira and Masaka study, our study sampled pigs originating from a wider geographic area over a longer period (13 months of sampling), hence giving us a more comprehensive picture of the seroprevalence of these pathogens in pigs at Kampala metropolitan area abattoirs. A recent cross-sectional study conducted in Lira, northern Uganda found a PRRSV seroprevalence of 7.5% (24), and a prevalence of 24.65% for PRRSV type 1 and 2.73% for PRRSV type 2 using molecular techniques (25).

In the present study we found a *Salmonella* spp. prevalence of 4.4% (40/903) among all pigs tested for *Salmonella* and 4.6% (26/568) among ASFV qPCR positive pigs tested for *Salmonella* spp. Furthermore, of the 40 *Salmonella* positive pigs, 65% (26/40) were ASFV qPCR positive. Our findings on *Salmonella* spp. prevalence are similar to those of a previous study (26) that found a 4% *Salmonella* spp. prevalence in swine fecal samples collected from Wambizi pig abattoir in Kampala. However, other previous studies in Uganda found higher prevalence of *Salmonella* spp. One study conducted on samples collected at Wambizi abattoir found a prevalence of 16.5% (33/100) in pig fecal matter and 10.5% (21/100) in muscle tissue (27). Another previous study conducted in 2011 and 2012 in northern and Eastern Uganda found an overall *Salmonella* spp. prevalence of 12% in suckling and weaned pigs (28). Although our findings show a relatively low occurrence of *Salmonella* spp. in slaughtered pigs, we were not able to culture and enrich samples before testing as was done as in these studies cited here and in other previous work (29). Salmonellosis needs to be considered an important differential diagnosis for ASF in Uganda that should be ruled out during ASF surveillance. Additionally, the reason 26/40 *Salmonella* positive pigs were ASFV qPCR positive is perhaps due to secondary *Salmonella* infections due to immunosuppression following ASFV infection. However, this needs to be further investigated.

In the present study, all the samples tested were negative for classical swine fever virus (CSFV), suggesting that CSFV is not circulating in Uganda. A previous study conducted in 2010–2011 that evaluated 239 pig samples found no CSFV positive pigs (30), which was similar to our results. This is an important finding in that efforts should be made to prevent the introduction of CSFV to Uganda through importation of pork products and illegal entry of pork products from endemic areas. An introduction of CSFV to Uganda would further cripple the pig industry that is struggling to contain ASFV. Since CSFV infection resembles ASFV infection, it is important to support any findings with diagnostics to ensure a definitive diagnosis is reached and a new disease is not missed during investigations.

This study had some limitations. It is possible that some misclassification bias was introduced during the qualitative analysis of free text entries in the dataset. However, such bias if any, is minimal because the qualitative analyses were performed by veterinarians and epidemiologists in the research team (JE and KH) who are knowledgeable of the clinical and pathologic presentation of ASF and in the analysis of qualitative data. It was necessary to include free-text entries into the abattoir data collection form to allow for the collection of clinical and pathologic information that would otherwise have been missed if it did not fit into pre-determined clinical signs and pathologic lesions severity categories. Also, the proportion of ASFV positive pigs with skin discolorations may be over-represented because some of the reddening observed could have resulted from insect/bug bites, and pre-slaughter pig restraint methods used at the abattoirs such as twisting of the ears and tails, dragging of pigs on the abattoir floors. It was difficult to observe skin discolorations in black/dark colored pigs.

## 4 Conclusion

ASF in pigs slaughtered in central Uganda presents with no clinical signs or pathologic lesions or with clinical signs and lesions typical of subacute to acute disease. It is not clear if the normal pigs are pre-clinical or have subclinical disease. Nonetheless, pig abattoirs in Uganda could be used as surveillance sites for identifying ASF outbreaks and pathologic lesions seem more reliable for syndromic surveillance than clinical signs. There is a high-level of exposure of pigs to S-IAV, a very low exposure to PRRSV, a relatively low number of pigs with detectable *Salmonella* spp., and no pigs with detectable CSFV among pigs slaughtered in and around Kampala. Due to the occurrence of other pathogens causing similar clinical signs and lesions, ASF surveillance programs in Uganda will require confirmatory laboratory diagnosis. There is no evidence that CSFV is currently circulating in Uganda and is not a differential diagnosis of concern at this time, but diagnostic testing to confirm suspect cases should be done to properly diagnose hemorrhagic disease cases in pigs and to identify an introduction should it occur.

## 5 Methods

### 5.1 Training of research assistants, and abattoir data collection form

Prior to data collection and sampling, a team of research assistants that comprised veterinarians and laboratory technologists were provided with a tailored manual (Supplementary file 2) on African swine fever, its clinical signs and lesions and the standard operating procedures for collecting blood and tissue samples for ASFV laboratory diagnosis. A veterinary pathologist familiar with ASF trained the research assistants on ASF and appropriate methods for sample collection, handling, and storage using the training manual as a guide. An abattoir data collection form (Supplementary file 3) that captured the clinical signs and lesions of the sampled pigs was developed and checked for validity and reliability by a team of veterinary pathologists in Uganda and the United States who are known to be experts in ASF. The abattoir data collection form was further modified to ease data capture (Supplementary file 4).

The abattoir data collection form had three sections: pig biodata, clinical scoring, and pathological scoring. The pig biodata section captured pig breed type (local, exotic, mixed), pig sex, district of origin, among other variables, while the clinical scoring section captured pre-slaughter clinical findings such as rectal temperature, clinical signs such as depression, abnormal gait, diarrhea, vomiting, body condition, cough, skin reddening (cyanosis). The clinical scoring scheme used was a modification of previously described ASF clinical scores (31–34). ASF body condition scoring was as described previously (35). The pathologic scoring section captured gross pathological lesions observed post-mortem and included lung, kidney, spleen, lymph node lesions and other lesion types as described previously (6, 32, 36, 37).

Following their training, the research assistants pretested the data collection form at three pig abattoirs under the guidance of the veterinary pathologist. Adjustments were made to the data collection form based on the feedback received from the abattoir pretesting. The data collection form was further modified and reformatted after the initial data collection to capture the number of days arriving pigs spent at the abattoir and to ease data entry.

### 5.2 Sampling and data collection

**Figure 4:**
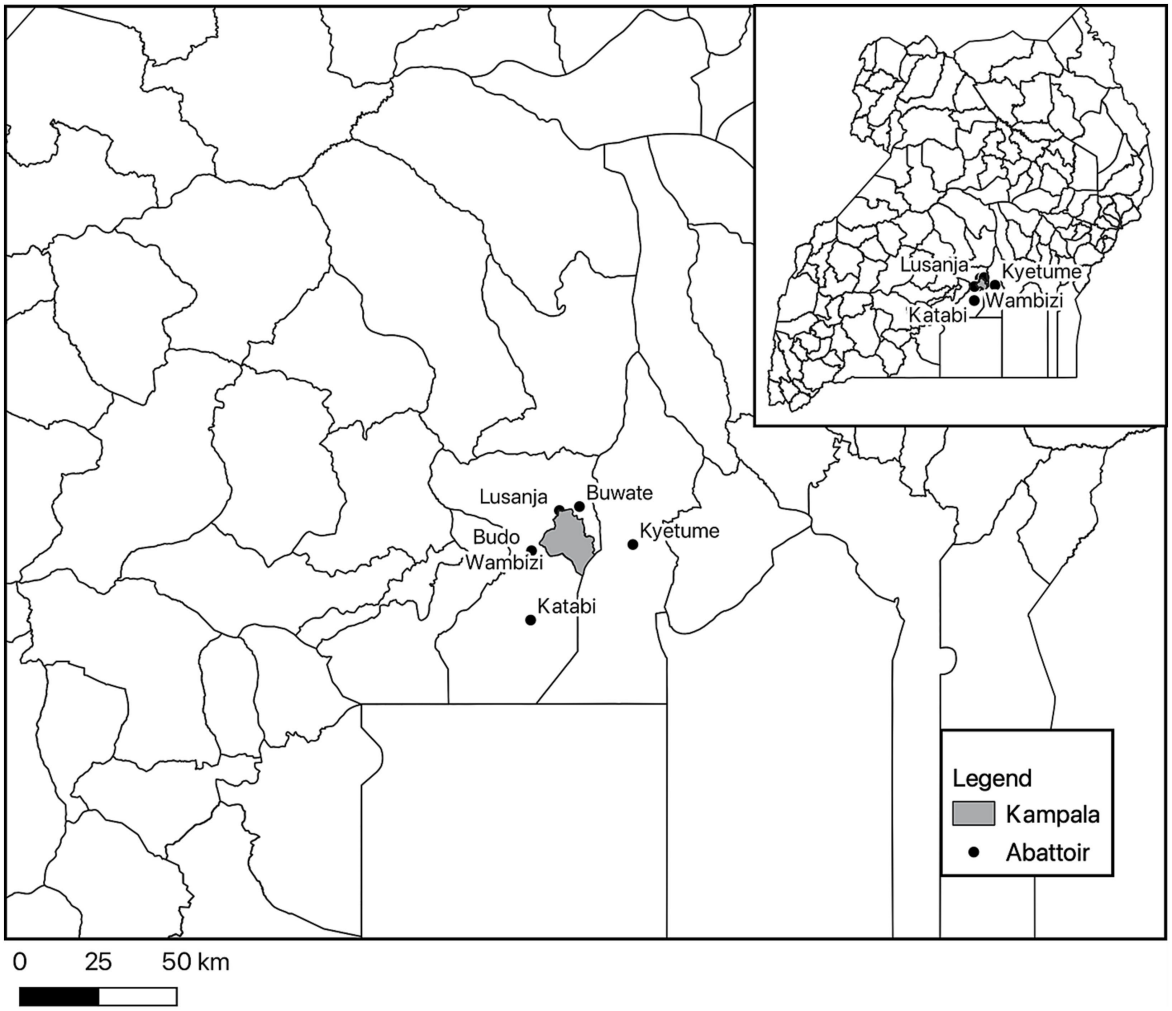
Map showing the location of the six abattoirs where pigs were sampled in the Kampala metropolitan area of Uganda.

Clinical and pathological data and samples were collected from Lusanja, Buwate, Kyetume, Budo, Katabi, and Wambizi pig abattoirs (Figure 4) located in the Kampala metropolitan area from May 2021 through June 2022. A stratified, systematic sampling approach that weighted sample sizes for each abattoir by the average annual slaughter rates was used. For purposes of systematic sampling, adjustments were made to the sample sizes in that monthly sample size was rounded to an even number if the determined monthly sample size was an odd number and a minimum sampling size per visit was set at four. Table 4 gives a summary of the monthly sampling frequency and associated sample sizes for each abattoir. Two to four sampling days were randomly selected each month for each abattoir. On the day of sampling, a systematic sampling approach was used to select pigs at the site until the desired sample size for that day was achieved. Typically, for each slaughterhouse visit, an estimate of the number of pigs to be slaughtered was determined and a sampling interval was computed by dividing the day’s estimated slaughter total by the required sample size for that visit.

**Table 4:** Distribution of the number for pigs sampled each month at six abattoirs in the Kampala metropolitan area, May 2021 through June 2022.

Abattoir	Planned # of pigs sampled per month	# visits per month	# of pigs sampled overall
Lusanja	36	4	443
Wambizi	32	4	396
Katabi	16	2	195
Budo	10	2	135
Buwate	8	2	103
Kyetume	8	2	62^a^

Samples per visit were the number of samples per month divided by the number of visits.

a Total samples collected was less than estimated due to COVID-19 restrictions and reduced slaughters at this abattoir.

At the study design stage, it was determined that 100 positive pigs were needed to reasonably characterize the clinical and pathologic presentation of ASF at the abattoirs. The prevalence of ASF was expected to be 11.5% (4) and the sample size needed to detect this prevalence at a 95% confidence level and 5% error rate was determined to be 157 pigs (openepi.com; Accessed July 2018). This would result in 18 positive pigs, and we sampled 1,200 pigs needed to ensure 100 positive pigs are sampled.

### 5.3 Confirmatory diagnostic testing for ASFV

Confirmatory diagnostic testing for ASFV was performed using the real-time PCR (qPCR) assay as we previously described (38). In brief, the samples tested included whole blood, pooled lymph nodes (submandibular, renal and gastro-hepatic), tonsil, and spleen. All samples were tested following standard operating procedures (SOPs) from the Foreign Animal Disease Diagnostic Laboratory (Plum Island Animal Disease Center, New York, United States), a World Organisation for Animal Health African swine fever virus reference laboratory. Only the clinical and pathologic lesions of qPCR positive pigs are described in this paper.

### 5.4 Diagnostic testing for ASFV differential diagnoses

#### 5.4.1 Serologic testing

Serum samples were prepared from blood collected into a clotting tube (Becton, Dickinson and Company, Franklin Lakes, New Jersey, United States). The tube was transported on ice to the diagnostic laboratory and stored at 4°C overnight to incubate. They were then centrifuged at 1,000 × *g* (Eppendorf 5804, Hamburg, Germany) for 10 min and then the serum was aliquoted into cryovials and stored at −20°C until testing occurred. Serum samples were tested for antibodies against swine influenza A virus (S-IAV) and porcine reproductive and respiratory syndrome virus (PRRSV). The INgezim PRRS 2.0 indirect ELISA for the detection of antibodies against North American and European PRRSV variants (Ingenasa, Madrid, Spain) and the indirect INgezim swine influenza 2.0 ELISA (Ingenasa, Madrid, Spain), which detects antibodies against influenza A viruses in swine, were used, and manufacturer’s instructions included in the kit were followed.

#### 5.4.2 Molecular testing

Pooled lymph nodes (submandibular, renal and gastro-hepatic) were tested for *Salmonella* spp. DNA, and tonsils for classical swine fever virus RNA. Tissue samples were stored at −20°C after collection until they were processed. Tissue processing occurred as follows. For each tissue type, one gram was washed in 1X phosphate buffered saline (PBS) solution (Thermo Fisher Scientific, Waltham, Massachusetts, United States) and homogenized using the Stomacher 80 Biomaster (Seward Ltd., West Sussex, United Kingdom). After the tissue was homogenized, 9 mL of 1X PBS was added and the mixture was centrifuged for 10 min at 1,000 × *g*. The supernatant was collected and stored at −20°C until extraction occurred. These procedures followed the US Department of Agriculture (USDA) Foreign Animal Disease Diagnostic Laboratory (FADDL) sample preparation standard operating procedures (SOPs) except that a stomacher was used rather than a tissue lyser for homogenization (39).

Lymph nodes underwent DNA extraction using the Qiagen DNeasy tissue and blood kit (Qiagen, Hilden, Germany) following the standard operating procedures developed by the USDA FADDL (39) and tonsils underwent RNA extraction using the Qiagen RNeasy mini kit (Qiagen, Hilden, Germany) also following the standard operating procedures developed by the USDA FADDL (40). Both DNA and RNA extraction aligned with the manufacturer’s instructions.

Real-time PCR (qPCR) assays were run on a Quantstudio 5 thermocycler (Thermo Fisher Scientific, Waltham, Massachusetts, United States). The *Salmonella* qPCR procedure has been previously described (29). The primers target highly conserved regions of the *Salmonella*-ttr locus (4). The samples were not enriched prior to testing. The forward primer of 5′-CTCACCAGGAGATTACAACATGG-3′, reverse primer of 5′-AGCTCAGACCAAAAGTGACCATC-3′, and a probe of 5′-FAM-CACCGACGGCGAGACCGACTTT-3′-BHQ1 (Eurofin Genomic, Munich, Germany) were used along with TaqMan Fast Virus 1-Step Master Mix (Thermo Fisher Scientific, Waltham, Massachusetts, United States). The VetMax Xeno DNA internal positive control (IPC) (Thermo Fisher Scientific, Waltham, Massachusetts, United States) was added to each sample prior to extraction and detected during qPCR using the VetMax Zeno IPC LIZ assay (Thermo Fisher Scientific, Waltham, Massachusetts, United States). CSFV detection was completed following USDA FADDL SOPs (41). The same master mix and VetMax Xeno Liz assay as was used for the *Salmonella* qPCR were used, except the VetMax Xeno IPC was an RNA control. The forward primer was 5′-TGCCCAAGACACACCTTAACC-3′, reverse primer was 5′-GGCCTCTGCAGCGCCCTAT-3′, and the probe was 5′-FAM-TGATGGGAGTACGACCTG-3′-MGBEQ (Eurofin Genomic, Munich, Germany). Not all tonsils and lymph nodes were tested due to a work stoppage implemented by the funding entity for political reasons.

### 5.5 Data management and analyses

Clinical and pathologic data captured in the data collection forms were entered into Microsoft Excel version 16 (Microsoft, Redmond, Washington, United States) and the data entry validated. Gross pathologic lesion data captured as free text was qualitatively assessed and re-aligned with existing score categories in the dataset where possible or discarded if they could not fit. The clinical signs and pathologic lesions data were collated in Microsoft Excel Version 2,306 Build 16.0.16529.20100 (Microsoft, Redmond, Washington, United States).

Diagnostic results were first evaluated by assessing the controls. For serology, this was the negative and positive controls and for molecular diagnostics this included negative and positive extraction and amplification controls as well as the IPC. Any results with failed controls were excluded. Any samples with a negative IPC and negative results were excluded. For any duplicate samples, results were compared across the CSFV or *Salmonella* spp. results as well as across ASFV results, if the results for the two pathogens agreed across the duplicate samples, the first sample tested was included. If the results differed, including Ct values with a difference >3 cycle threshold (Ct) values, then those samples were excluded. Data was collated in Microsoft Excel v 16.74 (Microsoft, Redmond, Washington, United States) in preparation for analysis.

Stata 18.0 and Stata 16.1 IC (Stata Corp, College Station, Texas, United States) were used for statistical analyses of the clinical signs and pathologic lesions and the differential diagnoses data, respectively. The clinical signs and gross pathologic lesion scores data were summarized using frequencies and proportions by sample type tested (blood, lymph nodes, tonsil, and spleen) and for all pigs that were ASFV positive by any of the sample types tested. The gross pathologic lesions were scored as described in Supplementary file 2, and the total sum of scores per pig sampled was calculated. The total possible severity score for the gross pathologic lesions was 33. Normality of the pathologic lesion scores data was evaluated using a visual assessment of a histogram and the Shapiro–Wilk test, and both showed the data were not normally distributed. Percentiles of the pathologic lesion scores were calculated. The Kruskal–Wallis test followed by Dunn’s test of multiple comparisons was used to evaluate for differences in median scores among the different sample types tested by qPCR and the level of significance for this test was 0.05, but influential variables were described as those with a probability value of less than 0.1. Percent positivity for the serologic and molecular results were summarized and 95% confidence intervals calculated using the Agresti-Coull method (42). The denominators used in the calculation of the proportions varied based on the completeness of the data for each variable in the dataset. The map showing the location of the pig abattoirs visited was created in QGIS Firenze version 3.28.1^1^ and the map shape file for Ugandan administrative districts were from the United Nations High Commissioner for Refugee Operations Data Portal (https://data.unhcr.org/en/documents/details/83043; Accessed March 30, 2023, published on November 17, 2020).

## Data Availability

The raw data supporting the conclusions of this article will be made available by the authors, without undue reservation.

## Footnotes

^1^ http://qgis.org
