# Supplementary material for: A description of the clinical signs and lesions of African swine fever, and its differential diagnoses in pigs slaughtered at selected abattoirs in central Uganda
Source: Front Vet Sci. 2025 May 29;12:1568095. doi: 10.3389/fvets.2025.1568095 (PMC12159021; doi:10.3389/fvets.2025.1568095)
Supplement: Supplementary file 1 [file Table_1.pdf]

Supplementary file 1

A description of the clinical signs and lesions of African swine fever, and its differential diagnoses in pigs slaughtered at selected abattoirs in central Uganda

John E. Ekakoro, Aisha Nassali, Cole Hauser, Krista Ochoa, Dickson Ndoboli, Rodney Okwasiimire, Edrine B. Kayaga, Eddie M. Wampande and Karyn A. Havas

***Correspondence: ekakoro@rowan.edu**

Table S1: Distribution of clinical signs based on sample type used to detect ASFV nucleic acid from pigs sampled from May 2021 through June 2022 from six abattoirs located in the Kampala metropolitan area of Uganda.

	**Sample type tested for ASFV qPCR diagnosis**				
	**Blood**	**Lymph nodes**	**Tonsil**	**Spleen**	**Overall***
	**Number (%)**				
**Depression/liveliness**	**n = 201**	**n = 452**	**n = 473**	**n = 395**	**n = 793**
Appeared normal with no evidence of depression	185 (92)	434 (96)	449 (94.9)	379 (95.9)	765 (96.5)
Pig was quiet and less responsive to its environment	12 (6)	13 (2.9)	18 (3.8)	11 (2.8)	20 (2.5)
The pig was moribund	4 (2)	5 (1.1)	6 (1.3)	5 (1.3)	8 (1)
**Gait**	**n = 196**	**n = 447**	**n = 466**	**n = 393**	**n = 785**
Pig had well-coordinated movements	182 (92.9)	426 (95.3)	441 (94.6)	374 (95.2)	753 (95.9)

Was hesitant to walk	6 (3.1)	7 (1.6)	11 (2.4)	8 (2)	11 (1.4)
A distinct staggering gait/hind lameness, but able to walk	3 (1.5)	6 (1.3)	6 (1.3)	3 (0.8)	7 (0.9)
Had serious lameness, unable to walk	1 (0.5)	1 (0.2)	1 (0.2)	2 (0.5)	2 (0.3)
Pig gait not observed	4 (2)	7 (1.6)	7 (1.5)	6 (1.5)	12 (1.5)
**Diarrhea**	**n = 201**	**n = 452**	**n = 473**	**n = 394**	**n = 792**
No diarrhea	198 (98.5)	446 (98.7)	466 (98.5)	386 (98)	781 (98.6)
Non-bloody diarrhea/evidence of non-bloody diarrhea	2 (1)	5 (1.1)	6 (1.3)	7 (1.8)	10 (1.3)
Bloody diarrhea/evidence of bloody diarrhea	1 (0.5)	1 (0.2)	1 (0.2)	1 (0.2)	1 (0.1)
**Vomiting**	**n = 200**	**n = 451**	**n = 472**	**n = 393**	**n = 792**
No vomiting/there was no evidence of vomiting	200 (100)	451 (100)	472 (100)	393 (100)	792 (100)
**Fever**	**n = 201**	**n = 453**	**n = 474**	**n = 395**	**n = 794**
No fever (rectal temperature within normal range)	78 (38.1)	167 (36.9)	189 (39.9)	161 (40.8)	319 (40.1)
Mild fever	32 (15.9)	57 (12.6)	56 (11.8)	47 (11.9)	81 (10.2)
Moderate fever	17 (8.5)	19 (4.2)	23 (4.9)	19 (4.8)	29 (3.7)
Severe fever	10 (5)	16 (3.5)	18 (3.8)	18 (4.6)	26 (3.3)
Rectal temperature below normal threshold*	57 (28.4)	185 (40.8)	178 (37.5)	142 (35.9)	326 (41.1)
Undetermined	7 (3.5)	9 (2)	10 (2.1)	8 (2)	13 (1.6)

Body condition	n = 197	n = 444	n = 468	n = 391	n = 785
Normal or over conditioned	158 (80.2)	381 (85.8)	398 (85)	337 (86.2)	680 (86.6)
Thin. Depression between the hip and vertebrae could be seen, pressure was required to feel bones on palpation	32 (16.2)	50 (11.3)	60 (13)	45 (11.5)	87 (11.1)
Emaciated. Hip and vertebral bones were prominent and easy to feel on palpation	7 (3.6)	13 (2.9)	10 (2)	9 (2.3)	18 (2.3)

Table S1 (Continued): Distribution of the clinical signs by sample type in ASFV positive pigs sampled between May 2021 and June 2022 from six abattoirs located in the Kampala metropolitan area of Uganda.

	Sample type				
	**Blood**	**Lymph nodes**	**Tonsil**	**Spleen**	**Overall***
**Clinical sign/antemortem picture**	**Number (%)**				
**Joints**	**n = 194**	**n = 441**	**n = 464**	**n = 385**	**n = 779**
Joint(s) normal	194 (100)	440 (99.8)	262 (99.6)	385 (100)	777 (99.7)
Severe joint swelling and lameness	0 (0)	1 (0.2)	2 (0.4)	0 (0)	2 (0.3)
**Breathing**	**n = 198**	**n = 448**	**n = 470**	**n = 393**	**n = 789**
Had a normal breathing pattern	194 (98)	444 (99.1)	463 (98.5)	388 (98.7)	781 (99)
Had labored breathing	4 (2)	4 (0.9)	7 (1.5)	5 (1.3)	1 (1)
**Cough**	**n = 199**	**n = 450**	**n = 470**	**n = 393**	**n = 790**

Not coughing	199(100)	450 (100)	470 (100)	393 (100)	790 (100)
**Nasal discharges**	**n = 196**	**n = 447**	**n = 468**	**n = 392**	**n = 788**
Had no nasal discharges	195 (99.5)	445 (99.5)	467 (99.8)	390 (99.5)	786 (99.8)
Had serous or seromucous nasal discharges	1 (0.5)	2 (0.5)	1 (0.2)	2 (0.5)	2 (0.2)
**Skin discoloration: cyanosis/hyperemia/hemorrhages of the skin**	**n = 184**	**n = 423**	**n = 435**	**n = 367**	**n = 738**
Absent (normal)	126 (68.5)	328 (77.5)	325 (74.7)	269 (73.3)	571 (77.4)
Present	58 (31.5)	95 (22.5)	110 (25.3)	98 (26.7)	167 (22.6)
**Skin necrosis**	**n = 198**	**n = 444**	**n = 465**	**n = 389**	**n = 781**
Had no signs of skin necrosis	192 (97)	436 (98.2)	458 (98.5)	383 (98.4)	771 (98.7)
Had raised reddened circular skin lesions	1 (0.5)	2 (0.5)	1 (0.2)	1 (0.3)	3 (0.4)
Had necrotic circular skin lesions	5 (2.5)	6 (1.3)	6 (1.3)	5 (1.3)	7 (0.9)
**Eyes/conjunctiva**	**n = 198**	**n = 447**	**n = 468**	**n = 393**	**n = 787**
Normal (light pink conjunctiva)	198 (100)	446 (99.8)	465 (99.4)	392 (99.8)	783 (99.5)
Reddened, clear secretion	0 (0)	1 (0.2)	3 (0.6)	1 (0.2)	4 (0.5)

*Overall includes all pigs that were ASFV positive by any of the sample types tested.

Table S2: Distribution of gross pathologic lesions found in ASFV-positive pigs sampled from May 2021 through June 2022 from six abattoirs located in the Kampala metropolitan area of Uganda.

Gross pathologic observations in organ/tissue	Lesion description	Lesion severity score	Sample type				
			Blood	Lymph nodes	Tonsil	Spleen	Overall*
			Number (Percentage)				
**Lungs**							
Lack of collapse			**n = 200**	**n = 452**	**n = 473**	**n = 393**	**n = 792**
	No rib impressions.	0	197 (98.5)	447 (98.9)	469 (99.2)	390 (99.2)	787 (99.4)
	Mild rib impressions.	1	3 (1.5)	3 (0.7)	3 (0.6)	3 (0.8)	3 (0.4)
	Clear rib impressions.	2		2 (0.4)	1 (0.2)	0 (0)	2 (0.2)
Edema			**n = 198**	**n = 449**	**n = 470**	**n = 392**	**n = 789**
	No apparent lung edema or froth in trachea.	0	71 (35.9)	145 (32.1)	143 (30.4)	105 (26.8)	225 (28.5)
	The lung had a moist and shiny appearance. Some froth in the trachea. Interlobular divisions are evident.	1	62 (31.3)	172 (38.3)	199 (42.3)	152 (38.8)	315 (39.9)
	The lungs are heavy. A lot of froth in the trachea. Interlobular divisions are marked.	2	65 (32.8)	132 (29.4)	128 (27.2)	135 (34.4)	249 (31.6)
Hemorrhage			**n = 200**	**n = 452**	**n = 472)**	**n = 394**	**n = 792**
	No hemorrhages observed.	0	132 (66)	289 (63.9)	310 (65.7)	253 (64.2)	511 (64.5)
	Patches of reddened tissue seen throughout the lungs.	1	43 (21.5)	114 (25.2)	115 (24.4)	102 (25.9)	210 (26.5)
	Widespread reddened section of lung with evidence of hemorrhage.	2	25 (12.5	49 (10.8)	47 (9.9)	39 (9.9)	71 (9)
Cranio-ventral Consolidation			**n = 187)**	**n = 421**	**n = 441**	**n = 377**	**n = 752**
	No consolidation.	0	182 (97.3)	415 (98.6)	435 (98.6)	372 (98.7)	745 (99.1)
	Mild consolidation in one/both lungs.	1	2 (1.1)	3 (0.7)	3 (0.7)	2 (0.5)	4 (0.5)
	Severe consolidation in one/both lungs.	2	3 (1.6)	3 (0.7)	3 (0.7)	3 (0.8)	3 (0.4)

*Overall includes all pigs that were ASFV positive by any of the sample types tested.

Table S2 (Continued): Distribution of gross pathologic lesions found in ASFV-positive pigs sampled from May 2021 through June 2022 from six abattoirs located in the Kampala metropolitan area of Uganda.

Gross pathologic observations in organ/tissue	Lesion description	Lesion severity score	Sample type				
			Blood	Lymph nodes	Tonsil	Spleen	Overall*
			Number (Percentage)				
**Kidneys**							
Hemorrhages			**n = 201**	**n = 448**	**n = 469**	**n = 393**	**n = 787**
	No hemorrhages on kidney surface.	0	74 (36.8)	157 (35)	157 (33.5)	118 (30)	260 (33)
	Some pinpoint (petechial) hemorrhages evident on kidney surface.	1	81 (40.3)	191 (42.6)	216 (46)	177 (45)	357 (45.4)
	Petechiation that is widespread on the surface of the kidney.	2	46 (22.9)	100 (22.3)	96 (20.5)	98 (25)	170 (21.6)
**Spleen**							
Enlargement			**n = 200**	**n = 451**	**n = 473**	**n = 392**	**n = 790**
	Spleen is of normal size.	0	108 (54)	288 (63.9)	294 (62.2)	227 (57.9)	510 (64.6)
	Mild to moderately enlarged spleen. Splenic capsule shows some cracking when spleen bent.	1	61 (30.5)	125 (27.7)	135 (28.5)	123 (31.4)	220 (27.8)
	Spleen is markedly enlarged (gigantic). Spleen breaks easily when bent.	2	31 (15.5)	38 (8.4)	44 (9.3)	42 (10.7)	60 (7.6)
Haemorrhagic			**n = 196**	**n = 442**	**n = 464**	**n = 385**	**n = 776**
	No observable changes in spleen colour.	0	84 (42.9)	220 (49.8)	216 (46.5)	166 (43.1)	377 (48.6)
	Multiple areas (patches) of the spleen are dark coloured, other parts are normal.	1	61 (31.1)	155 (35	176 (38)	158 (41)	304 (39.2)
	The entire spleen is very dark red (almost black).	2	51 (26)	67 (15.2)	72 (15.5)	61 (15.8)	95 (12.2)

*Overall includes all pigs that were ASFV positive by any of the sample types tested.

Table S2 (Continued): Distribution of gross pathologic lesions found in ASFV-positive pigs sampled from May 2021 through June 2022 from six abattoirs located in the Kampala metropolitan area of Uganda.

Gross pathologic observations in organ/tissue	Lesion description	Lesion severity score	Sample type				
			Blood	Lymph nodes	Tonsil	Spleen	Overall*
			Number (Percentage)				
**Pericardial cavity**							
Hydropericardium			**n = 169**	**n = 349**	**n = 367**	**n = 293**	**n = 603**
	No fluid in the pericardial cavity.	0	141 (83.4)	285 (81.7)	293 (79.8)	231 (78.8)	471 (78.1)
	Straw-coloured fluid in pericardial cavity.	1	21 (12.4)	49 (14)	58 (15.8)	50 (17.1)	106 (17.6)
	Blood-stained fluid in pericardial cavity.	2	7 (4.1)	15 (4.3)	16 (4.4)	12 (4.1)	26 (4.3)
Fibrinous pericarditis			**n = 198**	**n = 444**	**n = 465**	**n = 391**	**n = 783**
	No fibrinous (yellowish) exudate.	0	195 (98.5)	442 (99.5)	463 (99.6)	389 (99.5)	779 (99.5)
	Fibrinous (yellowish) exudate observed.	1	3 (1.5)	2 (0.5)	2 (0.4)	2 (0.5)	4 (0.5)

*Overall includes all pigs that were ASFV positive by any of the sample types tested.

Table S2 (Continued): Distribution of gross pathologic lesions found in ASFV-positive pigs sampled from May 2021 through June 2022 from six abattoirs located in the Kampala metropolitan area of Uganda.

Gross pathologic observations in organ/tissue	Lesion description	Lesion severity score	Sample type				
			Blood	Lymph nodes	Tonsil	Spleen	Overall*
			Number (Percentage)				
**Lymph nodes**							
Hepato-gastric lymph nodes			**n = 197**	**n = 443**	**n = 468**	**n = 385**	**n = 781**
	No hemorrhage/edema.	0	82 (41.6)	210 (47.4)	210 (44.9)	147 (38.2)	345 (44.2)
	Enlarged and edematous.	1	23 (11.7)	52 (11.7)	61 (13)	52 (13.5)	107 (13.7)
	Multifocal hemorrhages with a marbled appearance.	2	41 (20.8)	106 (23.9)	118 (25.2)	111 (28.8)	217 (27.8)
	Enlarged lymph node with diffused hemorrhage.	3	51 (25.9)	75 (16.9)	79 (16.9)	75 (19.5)	112 (14.3)
Renal lymph nodes			**n = 197**	**n = 447**	**n = 464**	**n = 388**	**n = 781**
	No hemorrhage/edema	0	77 (39.1)	205 (45.9)	210 (45.3)	156 (40.2)	348 (44.6)
	Enlarged and edematous	1	25 (12.7)	49 (11)	61 (13.2)	51 (13.1)	97 (12.4)
	Multifocal hemorrhages with a marbled appearance.	2	37 (18.8)	97 (21.7)	95 (20.5)	97 (25)	192 (24.6)
	Enlarged lymph node with diffused hemorrhage.	3	58 (29.4)	96 (21.5)	98 (21)	84 (21.7)	144 (18.4)
Submandibular lymph nodes			**n = 199**	**n = 447**	**n = 470**	**n = 391**	**n = 787**
	No hemorrhage/edema.	0	131 (65.8)	312 (69.8)	325 (69.1)	264 (67.5)	555 (70.5)
	Enlarged and edematous.	1	33 (16.6)	72 (16.1)	79 (16.8)	75 (19.2)	134 (17)
	Multifocal hemorrhages with a marbled appearance.	2	17 (8.5)	41 (9.2)	45 (9.6)	29 (7.4)	70 (8.9)
	Enlarged lymph nodes with diffused hemorrhage.	3	18 (9.1)	22 (4.9)	21 (4.5)	23 (5.9)	28 (3.6)
Mesenteric lymph nodes			**n = 196**	**n = 439**	**n = 460**	**n = 384**	**n = 773**
	No hemorrhage/edema.	0	144 (73.5)	363 (82.7)	369 (80.2)	308 (80.2)	647 (83.7)
	Enlarged and edematous.	1	9 (4.6)	22 (5)	30 (6.5)	23 (6)	45 (5.8)
	Multifocal hemorrhages with a marbled appearance.	2	22 (11.2)	32 (7.3)	36 (7.8)	31 (8.1)	54 (7)
	Enlarged lymph nodes with diffused hemorrhage.	3	21 (10.7)	22 (5)	25 (5.4)	22 (5.7)	27 (3.5)

*Overall includes all pigs that were ASFV positive by any of the sample types tested.

Table S2 (Continued): Distribution of gross pathologic lesions found in ASFV-positive pigs sampled from May 2021 through June 2022 from six abattoirs located in the Kampala metropolitan area of Uganda.

Gross pathologic observations in organ/tissue	Lesion description	Lesion severity score	Sample type				
			Blood	Lymph nodes	Tonsil	Spleen	Overall*
			Number (Percentage)				
**Other organs with hemorrhages (excluding kidneys, lungs, spleen, lymph nodes)**							
Intestinal serosa/outside surface of intestines			**n = 200**	**n = 448**	**n = 468**	**n = 390**	**n = 785**
	No hemorrhage.	0	185 (92.5)	421 (94)	437 (93.4)	362 (92.8)	740 (94.3)
	Hemorrhage present.	1	15 (7.5)	27 (6)	31 (6.6)	28 (7.2)	45 (5.7)
Urinary bladder serosa/outside surface of the urinary bladder			**n = 174**	**n = 400**	**n = 415**	**n = 349**	**n = 697**
	No hemorrhage.	0	171 (98.3)	396 (99)	412 (99.3)	346 (99.1)	691 (99.1)
	Hemorrhage Present.	1	3 (1.7)	4 (1)	3 (0.7)	3 (0.9)	6 (0.9)
Pericardium/epicardium			**n = 188**	**n = 426**	**n = 444**	**n = 377**	**n = 754**
	No hemorrhage.	0	186 (98.9)	424 (99.5)	442 (99.5)	373 (98.9	750 (99.5)
	Hemorrhage present.	1	2 (1.1)	2 (0.5)	2 (0.5)	4 (1.1)	4 (0.5)
Renal fat			**n = 197**	**n = 446**	**n = 469**	**n = 390**	**n = 786**
	No edema.	0	194 (98.5)	438 (98.2)	463 (98.7)	386 (99)	775 (98.6)
	Edema present.	1	3 (1.5)	8 (1.8)	6 (1.3)	4 (1)	11 (1.4)
Gall bladder			**n = 176**	**n = 391**	**n = 422**	**n = 340**	**n = 689**
	No edema.	0	174 (98.9)	390 (99.7)	419 (99.3)	338 (99.4)	686 (99.6)
	Edema present.	1	2 (1.1)	1 (0.3)	3 (0.7)	2 (0.6)	3 (0.4)
**Other organs/tissues where edema or necrosis was present**							
Tongue			**n = 192**	**n = 431**	**n = 454**	**n = 384**	**n = 768**
	No evidence of necrosis.	0	192 (100)	431 (100)	454 (100)	384 (100)	768 (100)
	Multifocal and whitish areas observed (necrosis present).	1	0 (0)	0 (0)	0 (0)	0 (0)	0 (0)
Tonsils			**n = 196**	**n = 444**	**n = 463**	**n = 384**	**n = 778**
	No abnormality observed.	0	179 (91)	427 (96.2)	446 (96.3)	366 (95.3)	758 (97.4)
	Multifocal and whitish areas observed in tonsils (necrosis present).	1	4 (2)	2 (0.4)	3 (0.7)	4 (1)	5 (0.6)
	The tonsil is red/purple, and/or marked exudate observed.	2	13 (7)	15 (3.4)	14 (3)	14 (3.7)	15 (2)

*Overall includes all pigs that were ASFV positive by any of the sample types tested.
