# Supplementary material for: A description of the clinical signs and lesions of African swine fever, and its differential diagnoses in pigs slaughtered at selected abattoirs in central Uganda
Source: Front Vet Sci. 2025 May 29;12:1568095. doi: 10.3389/fvets.2025.1568095 (PMC12159021; doi:10.3389/fvets.2025.1568095)
Supplement: Supplementary file 2 [file Data_Sheet_1.pdf]

An evaluation of African swine fever presentation and distribution in Uganda

Project training manual for field data and sample collection

2020-2021

Cornell University

School of Veterinary Medicine & Animal Resources
Makerere University

Table of Contents

Project team 3

Introduction 4

Overall project objective 4

Study methods and procedures 4

An overview of African Swine fever 7

General standard operating procedures (SOPs) for quality sample collection and handling .. 12

Standard operating procedures for pig restraint and for blood collection 14

Clinical/antemortem inspection 16

Specific standard operating procedures for tissue collection 20

Postmortem inspection 25

Data collection tools 32

Funding 33

Acknowledgements 33

Photo Sources 33

References 33

Appendix: Abattoir Data collection form 35

Project team

Dr. Karyn Havas (Principal Investigator) – Cornell University, Ithaca New York, USA.

Dr. John Eddie Ekakoro (Lead author) – Cornell University, Ithaca, New York, USA.

Dr. Eddie Wampande – Makerere University, Kampala Uganda.

Mr. Dickson Ndoboli – Makerere University, Kampala Uganda.

Dr. Deo Ndumu – Ministry of Agriculture, Animal Industry and Fisheries, Uganda.

Dr. Julius Lutwama – Uganda Virus Research Institute.

Dr. Hector Aguilar-Carreno – Cornell University, Ithaca New York, USA.

Dr. Roger Barrette - U.S. Department of Agriculture Foreign Animal Disease Diagnostic Laboratory.

Introduction

This training manual is designed to prepare the reader to appropriately support the study through proper sample collection. It will orient you to the purpose of the study, evaluating the presentation and distribution of African swine fever in Uganda, and will provide the reader with an overview of African swine fever. Specifically, this training manual aims to:

a) To provide the project research assistants, veterinarians, slaughterhouse workers and laboratorians with a general overview of the project.
b) To provide the research assistants, veterinarians, slaughterhouse workers, and laboratorians with a general overview of African swine fever.
c) To describe the standard operating procedures for quality sample collection, and handling.
d) To train the research assistants on how to use the data collection tools.

Overall project objective

The overall objective of this project is to characterize the transmission, biosecurity, as well as the clinical and pathologic presentation of African swine fever (ASF) in market (slaughterhouse) swine around Entebbe and Kampala. The project will also correlate these findings to serologic and molecular diagnostic results, as well as sequencing results to better develop and enhance disease surveillance programs and diagnosis in Uganda. This training will basically focus on the clinical and pathologic presentation of ASF and on the standard operating procedures for quality sample collection and handling at the slaughterhouses.

Study methods and procedures

- The first part of this project will involve training research assistants, veterinarians, slaughterhouse workers and laboratorians on ASF clinical signs and

pathology, and laboratorians on ASF diagnostic assays prescribed by World Organization for Animal Health (OIE)[1].

- We will systematically sample domestic pigs at six slaughterhouses around Entebbe and Kampala. The six slaughterhouses are in the towns of Wambizzi, Matuga, Mukono, Entebbe, Luwero, and Kampala. It is important to note that environmental sampling (e.g., collection of abattoir run-off and effluent) will not be done. This is because the ASF virus persists a long time in the environment and environmental sampling will not be indicative of disease in the pigs being slaughtered that day and would not help inform tracebacks.
- At each slaughterhouse, sampled pigs will be scored based on clinical signs (antemortem) and pathology (postmortem) using scoring algorithms provided in this manual.
- Appropriate samples will be tested at Central Diagnostic Laboratory at Makerere University College of veterinary Medicine, Animal Resources and Biosecurity using OIE-prescribed assays with confirmatory testing at Ministry of Agriculture, Animal Industry and Fisheries' National Animal Disease Diagnostic and Epidemiology Center as needed.
- We will sample 200 pigs from each slaughterhouse for a total of 1200 pigs sampled over the course of the study. We expect that this will yield at least 120 ASF positive pigs. These numbers are based on epidemiologic sample size computation based on findings of past studies and will enable us to achieve the appropriate geographic, molecular, and clinical diversity of samples and will account for sample integrity and quality issues.
- Sampling will occur over the course of 12 months using biweekly (twice a month) sampling. We will sample 7-8 pigs every other week from each slaughterhouse. This biweekly sampling was decided based on resource and time constraints of the Makerere University students.
- Systematic sampling will be used for collection at each slaughterhouse. For example, if 120 pigs are being slaughtered on a given day, every 15th pig will be

assessed and sampled in order to sample 8 pigs. Animals will be given an ear tag ID during the antemortem exam to correctly identify them for the pathologic exam as well as to collect and label samples as the pig moves through the slaughter process.

- The clinical signs that will be evaluated include attitude, temperature, diarrhea, and vomiting, with and without blood, as well as hyperemic/hemorrhagic changes to the skin.
- We will not perform full necropsies at the abattoir but a targeted assessment of the organ pluck. The tissues to be collected will include the tonsils, spleen, gastro-hepatic lymph node, and mandibular lymph nodes.
- All pigs sampled will have their sex, breed, duration of ownership (if possible), and location of purchase prior to slaughter collected.
- Data collection and management will be standardized, and unique IDs, which will be the same as the ear tag numbers, will be used to link animal data to samples.
- The samples will be stored appropriately at Makerere University to ensure sample integrity. OIE-prescribed serologic and PCR assays in close accordance with quality standards defined by the International Organization for Standardization (ISO) 17025:2005 principles will be used.
- Diagnostics for ASF differential diagnoses (e.g., porcine reproductive and respiratory syndrome (PRRS), Influenza A, classical swine fever (CSF) and septicemic salmonellosis) will be completed as well.
- Upon completion of the study, a representative set of positive samples will be stored at NADDEC in their secure freezer space following an established standard operating procedure (SOP) for sample receipt and storage. Unnecessary samples will be destroyed by running them through the autoclave and incinerator at Makerere University prior to disposal.
- We plan to compare the clinical signs, pathology, serologic, and molecular (CPSM) diagnostic results using kappa scoring. This will evaluate the different disease presentation and diagnostic result combinations occurring in Uganda.

We will compare the ability of clinical scores, pathology scores, serologic results, and PCR results to classify an animal as positive for ASF.

- We plan to perform a pairwise comparison of the agreement between the four different commonly used ASF diagnostic tools (ASF Antibody detection ELISA, ASF antigen detection ELISA, ASF PCR, Indirect Immunoperoxidase test). We will adjust for bias and prevalence to provide an accurate kappa score.
- Pig demographic data/biodata, e.g., breed, sex, and region of origin, will be analyzed to control for confounding or association with the CPSM patterns to determine the probability of each pattern resulting in a pig being positive for ASF.
- All procedures and processes associated with sample collection from animals have been submitted to the US Department of Defense Animal Care and Use Review Office, the Cornell Institution Animal Care and Use Committee and the Research Ethics Committee at Makerere University's School of Veterinary Medicine and Animal Resources for review and approval.

An overview of African Swine fever

a) Etiology

African swine fever is a highly contagious hemorrhagic disease of swine caused by a large double-stranded DNA virus. It is an *asfivirus* commonly called African swine fever virus (ASFV). ASFV is the only known DNA arbovirus transmitted by soft ticks of the genus *Ornithodoros*[2,3]. ASFV has 24 distinct genotypes so far identified[4,5]. Genotype 1 is west African, and the other 23 circulate in eastern Africa. Genotype IX and X have been shown to circulate in Uganda[6].

b) Epidemiology

- ASF has a complex epidemiology with different patterns (sylvatic cycle, tick-pig cycle, and domestic cycle) which depend on the interrelationships and densities of the hosts and vector species[7].

- ASF is present in wild and/or domestic pigs in regions of Africa, Asia, and Europe. The Americas are currently ASF free.
- Disease transmission[8] occurs via;
    - Direct contact with infected swine (domestic or wild).
    - Indirect contact via ingestion of contaminated materials such as food waste, feed, or garbage, specifically feeding contaminated pork products to pigs.
    - In Africa, free-roaming pigs in villages contribute to disease spread.
    - Through fomites.
    - Transmission can occur from wildlife to domestic pigs through the bite of an infected soft tick (*Ornithodoros* ticks) or by ingestion of warthog tissues.

c) Clinical presentation/signs

- The clinical presentation of ASF varies depending on how virulent the infecting strain is.
- In domestic pigs, clinical ASF may take any of the following courses: peracute (hyperacute), acute, subacute, and chronic and mortality rates ranging from 0% to 100%[9].
- The clinical signs[8] may include fever, depression, huddling together (See **Figure 1a**), inappetence/mild anorexia, diarrhea (sometimes bloody), skin discoloration, skin necrosis in the chronic form, abortions in pregnant sows (due to the fever). Sudden death with no clinical signs may be reported (**Figure 1b**).

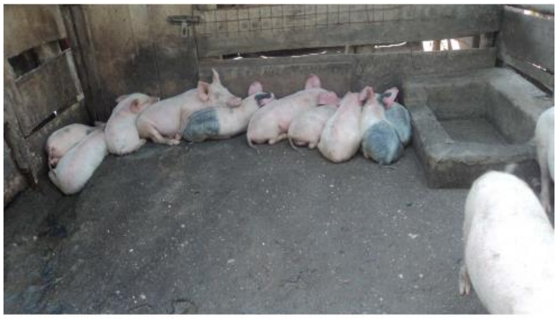

Figure 1a: Huddling in an ASF infected piggery unit in Kanyanya, Kawempe Division, Kampala.
Photo by Ekakoro, 2013.

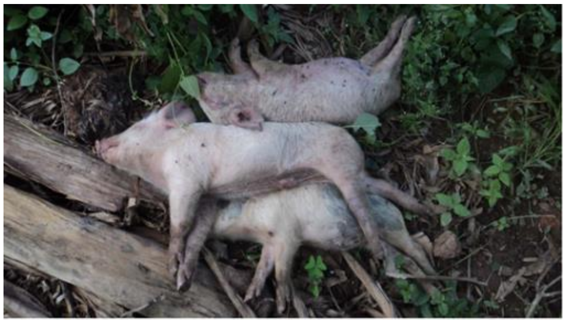

Figure 1b: Sudden death reported in Kanyanya, Kawempe Division, Kampala.
Photo by Ekakoro, 2013.

(More images on clinical signs are presented in the clinical/antemortem inspection section of this manual.)

d) Lesions

- The lesions observed vary depending on the virulence of the viral strain[2].
- For peracute ASF, no gross lesions are usually observed at postmortem (PM).
- The acute and subacute forms of the disease present with extensive hemorrhages and lymphoid tissue destruction. The most characteristic postmortem lesion of acute ASF is hemorrhagic splenomegaly, followed by multifocal hemorrhagic lymphadenitis.
- The subclinical and chronic forms of the disease may present with minimal or no lesions.

e) Diagnosis

- A definitive ASF diagnosis cannot be arrived at basing on clinical signs and or gross lesions due to its similarity to several other diseases like classical swine fever and PRRS. Laboratory studies are necessary for a definitive ASF diagnosis[10].
- Several laboratory tests are available for ASF diagnosis. The OIE describes several test methods that aim at either detecting an immune response or at identifying the agent (ASFV)[1].

Tests that aim at detecting an immune response include

I. Enzyme-linked immunosorbent assay (ELISA).
II. Indirect immunoperoxidase test (IPT). This is an OIE recommended method as a confirmatory serological test.
III. Indirect fluorescent antibody test (IFAT). This is also an OIE recommended confirmatory serologic test.
IV. Immunoblotting test (IBT).

Tests that aim at identifying the agent

I. Virus isolation/hemadsorption test.
II. Fluorescent antibody test (FAT).
III. Enzyme linked immunosorbent assay (ELISA) for antigen detection.
IV. Conventional polymerase chain reaction (PCR).
V. Real-time polymerase chain reaction (qPCR).

f) Differential diagnosis list[11]

- Classical swine fever.
- Swine erysipelas.
- Septicemic salmonellosis.
- Highly-pathogenic porcine reproductive and respiratory syndrome (HP-PPRS).
- Porcine dermatitis nephropathy syndrome (PDNS).
- Leptospirosis.
- Acute pasteurellosis.
- Trypanosomosis.
- Other hemorrhagic diseases/conditions of swine, e.g., hemorrhagic diathesis (bleeding disorders).
- Certain types of poisons, e.g., coumarin poisoning.

g) Public health implications

ASF does not directly affect humans. It is not a zoonotic disease. However, it indirectly affects humans since it is a serious threat to food security and has serious

social and economic impact on trade in swine and swine products/byproducts. ASF losses are indirectly associated with depression and poor health among pig producers[12].

h) Prevention and control

- No treatment or effective vaccine is currently available.
- Protection of ASF-free areas from the introduction of ASF is very important. This can be achieved through restriction of movement of infected live pigs and pig meat products[9].
- Strict bio-security protocols can be helpful in the prevention and control[13].
- Pig movement restriction and early detection of the disease through appropriate diagnostic testing is critical for prevention and control.
- In endemic areas, such as Sardinia, prevention relies on the control of pig movements and pig products in combination with extensive serological surveys to detect carriers.
- Successful eradication programs involve rapid diagnosis, slaughter, and disposal of all animals on infected premises, strict biosecurity, controlled movement etc.
- Control of the natural reservoirs in areas where they exist, e.g., sub-Saharan Africa, is important (*Ornithodoros* ticks) to prevent contact with domestic pigs.

General standard operating procedures (SOPs) for quality sample collection and handling

Follow the standard operating procedures (SOPs) mentioned below for quality sample collection and handling[14,15].

A. Prepare the sample collection supplies. Review and prepare your field kit before travelling to the abattoir for sample collection.
    a. Ensure adequate supplies for the number of animals to be sampled.
        i. Needles
        ii. Blood tubes
        iii. Whirl-pak or new plastic resealable bags – one for each tissue sample collected.
        iv. Data recording supplies
    b. Ensure you have working felt tip pens.
    c. Ensure you have cold boxes that seal and plug into a vehicle or with cold packs.
    d. Ensure that you carry enough forceps, scalpel blades and scalpel holders for each pig. Each pig sampled will need a separate scalpel blade mounted on scalpel holder and tissue forceps.
    e. Ensure you carry garbage bags for storing all the waste generated during the sampling.
B. All sample should be labeled using a smear proof or waterproof marker on a sticker or on the label area of the bag, but not on the clear plastic. The sample labeling should include:
    a) the pig ID ear tag number
    b) specific sample type
    c) date of collection
    d) abattoir name

C. The samples for this ASF investigation will include serum, whole blood, and fresh tissue samples. Collect whole blood in the 10 ml purple-top vacutainer tubes and serum in the 10 ml red-top vacutainer tubes.
D. The fresh tissue samples will include tonsils, spleen, gastro-hepatic lymph node, and mandibular lymph nodes.
E. Ensure appropriate personal protective equipment (PPE) for all involved in the sampling for protection from injuries and exposure to blood and other body fluids. The appropriate PPE will include overalls or plastic apron, gum boots, disposable gloves, and eye and ear protection. Hands should be washed, and gloves changed between animals.
F. Dispose of all sharp objects, e.g., needles and scalpel blades, in a leak-proof, puncture resistant sharps container. All reusable instruments, such as scalpel blade handles scissors, and forceps, should be used one time on one pig on the day of sampling and brought back to the laboratory to be thoroughly cleaned, disinfected, and autoclaved prior to reuse.
G. Place all the garbage that will be generated into the garbage bags provided and transport them back to the university for appropriate disposal. Do not dispose of the garbage at the slaughterhouse. All the garbage should be incinerated back at the university.
H. As a general principle, samples should never be submitted to the lab frozen, but should be refrigerated. Use cool boxes with ice packs for sample storage and transportation to the lab.
I. Ensure that you appropriately disinfect your gum boots after the sampling process at the slaughterhouse before traveling back to the laboratory. Place the disinfected gumboots in the boot (trunk) of the car and do not wear them in the car after disinfection.

Standard operating procedures for pig restraint and for blood collection

1. You will need syringes, needles, and vacutainer tubes. For small sized pigs (up to 25kg) use 20-gauge ¾-1-inch needles. For 25-75kg pigs, use 18 gauge, 1.5-inch needles, and for large pigs (>75kgs), use 14-16-gauge 1-2-inch needles (if collecting from the anterior vena cava, use a 4-inch length needle).
2. Proper restraint will be required for blood collection. Restraint the pig appropriately. Depending on how the abattoir situation presents, you can use either of the approaches mentioned below for restraining and collecting the blood samples.

   a) Dorsal recumbency

   Place the pig on dorsal recumbency with the head, fore and hind limbs appropriately restrained as shown in the image below (**Figure 2**).

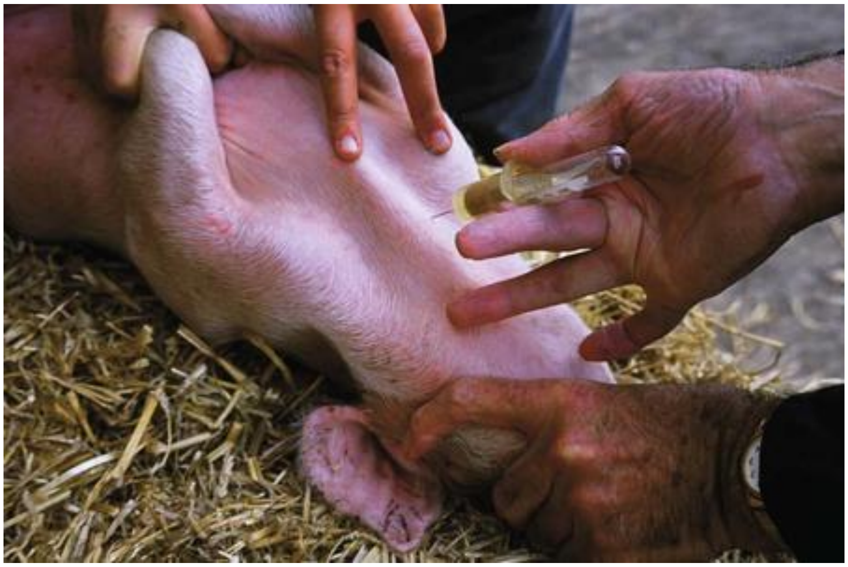

Figure 2: Blood sample collection with the pig in dorsal recumbency.
Photo source: https://veteriankey.com/porcine-clinical-procedures/

b) Using a snare

Restrain the pig with a snare securely contained against a wall as far back as possible and looped over the upper jaw and snout (**Figure 3, photos 1 and 2**). The handle should be held vertically. A pig should never be moved by pulling on a snare.

3. Clean the sample collection site as needed to remove any superficial dirt or debris.
4. Collecting from the jugular (**Figure 3, Photos 2 to 4**).
   i) Locate the right jugular furrow of the pig closer to the first rib than the jaw, with bevel up, insert needle perpendicular to the skin. If you missed the vein, carefully reposition the needle until you penetrate the vessel.
   ii) Once collection is complete, apply pressure over injection site and remove the needle. For adequate hemostasis, apply pressure for 30-60 seconds.
   iii) Dispose of the needle in the sharps or hard-sided plastic container.
5. Collecting from the anterior vena cava (**Figure 4**).
   iv) Use the same technique described for the jugular, and a needle of up to 4 inches in length.
   v) Insert the needle in the right jugular furrow just alongside cranial side of the first rib and then direct it slightly inwards towards the spine.
   vi) Insert the needle straight in and remove the needle slowly in the same manner to avoid damaging the vessel.

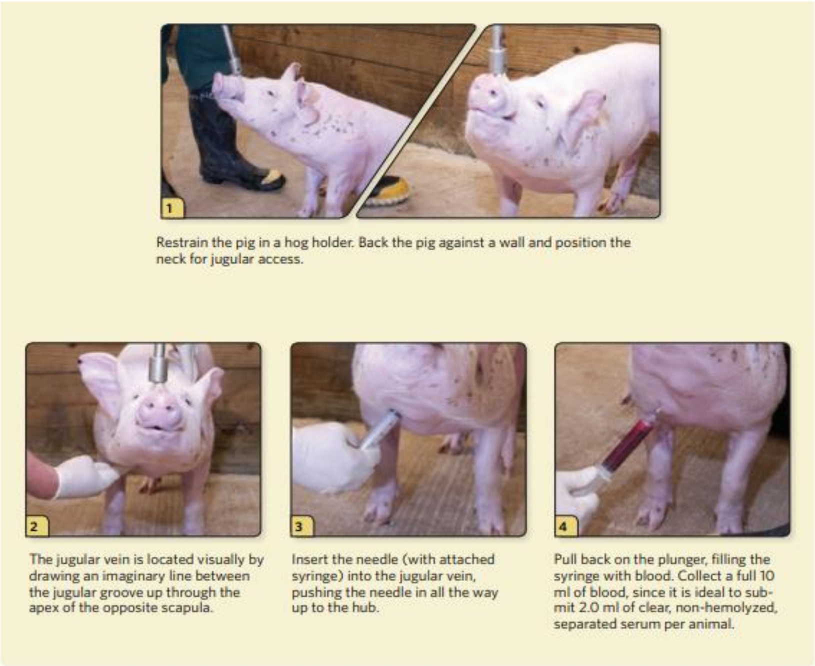

Figure 3: Collecting blood from the pig's jugular vein.

Extracted from the US Department of Agriculture, Foreign Animal Disease Investigation Manual, 2014: https://www.usdatraining.com/powerpoint_docs/FADD_Manual_digital.pdf.

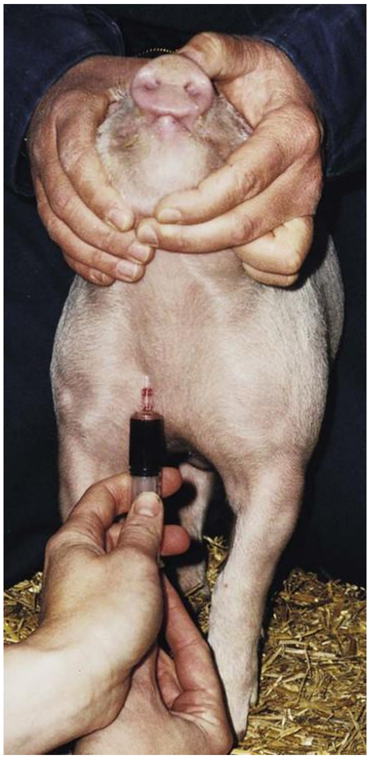

Figure 4: Collecting blood *from the anterior vena cava*

Photo source: https://veteriankey.com/porcine-clinical-procedures/

Clinical/antemortem inspection

In this part of the manual, we outline the standard operating procedures for clinical/antemortem inspection.

1. Ensure that you perform the antemortem inspection in an area with adequate lighting where the animal(s) will be properly observed.
2. During the antemortem inspection, look for the following:
    - The general behavior of the animal: Is the animal lively or depressed?
    - Abnormalities in gait: How is the animal walking? Is it circling, lame, or shifting its weight?
    - Abnormal discharges from the body orifices: Does the animal have diarrhea? If yes, is the diarrhea bloody? Is there any other evidence of diarrhea? Are there any nasal discharges?
    - Vomiting: Is the animal vomiting or is there evidence of any vomiting?

- Abnormal respiration: Is the breathing normal or is it labored? Is the animal coughing? Is it standing with its front legs wide apart, and its head extended trying to breath?
- Body condition: Is the animal emaciated or thin? Does the pig look normal or over-conditioned (fat or very fat)? See body condition scoring chart (**Figure 5**).
- Joint abnormalities: Are the joints visibly swollen?
- Skin abnormalities: Do you see any skin discoloration/hemorrhages particularly on the skin of the ears, nose, abdomen, flanks, legs, and tail? Do you see any necrotic skin lesions?
- Ocular/conjunctival abnormalities: Do you see any conjunctival reddening? Are there any discharges observed in the eyes? Are there squinting and/or teary eyes?
- Rectal temperature: Is the animal running a fever? What is the animal's rectal temperature? Note: If the thermometer returns bloody after taking the rectal temperature, do not conclude that the blood observed on the thermometer is evidence of bloody diarrhea. The mucosa can be very delicate when they are infected and the thermometer itself may have caused the bleeding. Be sure to lubricate the thermometer well before use.

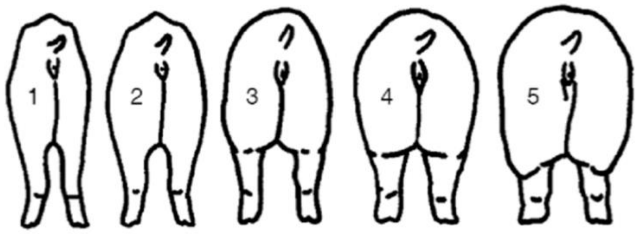

Score	Condition	Detection of ribs, backbone, "H" bones, and "pin" bones
1	Emaciated	Obvious
2	Thin	Easily detected with pressure
3	Ideal	Barely felt with firm pressure
4	Fat	None
5	Overly fat	None

Figure 5. Body condition scoring chart.

Source: Courtesy of Coffey et al,1999 (http://www2.ca.uky.edu/agcomm/pubs/asc/asc158/asc158.pdf).

3. The pictures below show signs of ASF that you should look out for during antemortem inspection. The pictures provided are not exhaustive of the ASF clinical signs. Here we present pictures of the most encountered clinical signs.

a) Hyperemia/cyanosis/hemorrhages of the skin

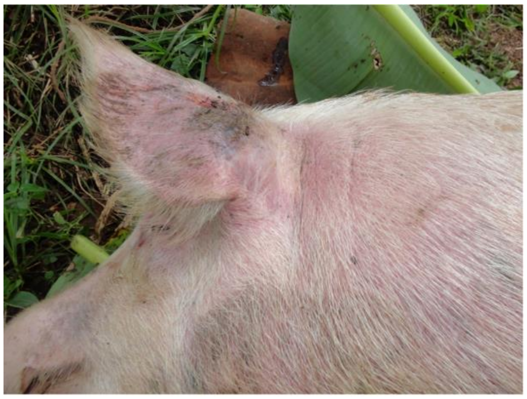

Figure 6a: Hyperemia of the skin of the ear.

Photo source: Ekakoro,2013.

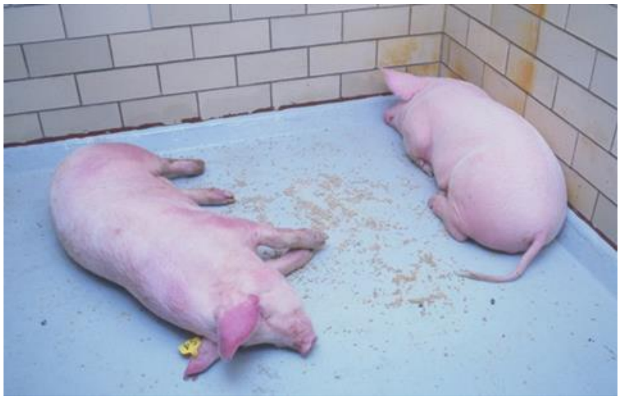

Figure 6b: Hyperemia, cyanosis, or hemorrhages of the skin of the ears, nose, abdomen, flanks, legs, and tail.

Photo credit: Plum Island Animal Disease Center.

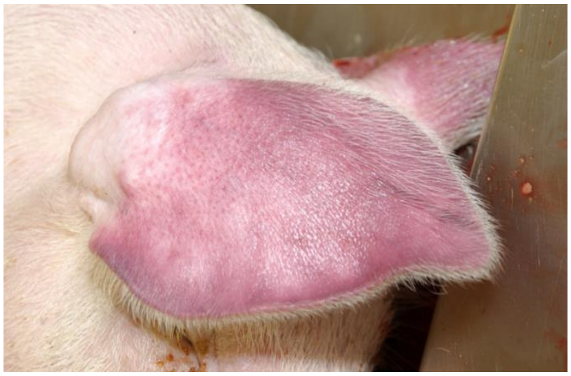

Figure 6c: Cyanosis of the skin of the ear.

Photo credit: Plum Island Animal Disease Center.

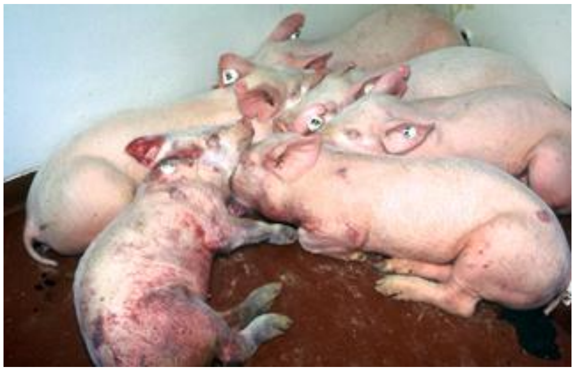

Figure 6d: Hemorrhages on the flanks and abdomen.

Photo credit: Plum Island Animal Disease Center.

b) Nasal discharge

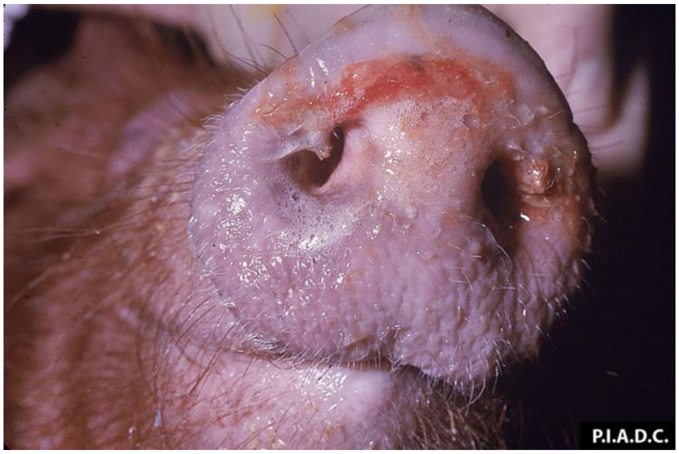

Figure 7: There is bloody, mucoid, foamy nasal discharge.

Photo credit: Plum Island Animal Disease Center and Iowa State University Center for Food Security and Public Health (CFSPH).

c) Discharges in the eyes.

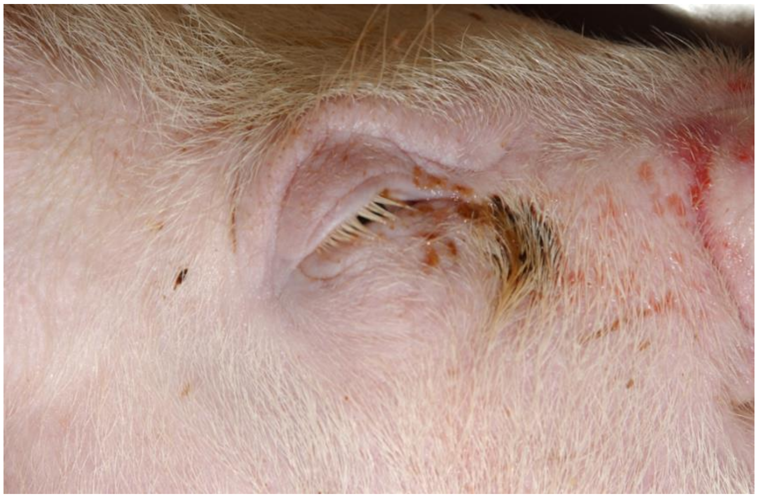

Figure 8: Ocular discharges, wet eye lashes, reddish discharge, and squinting eyes.

Photo source: Plum Island Animal Disease Center.

c) Necrotic skin lesions seen in chronic ASF

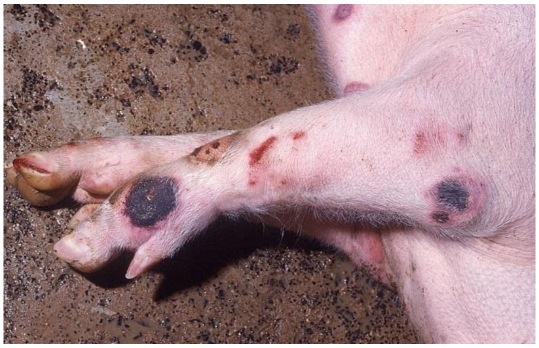

Figure 9a: Necrotic skin lesions.

Photo source: Plum Island Animal Disease Center.

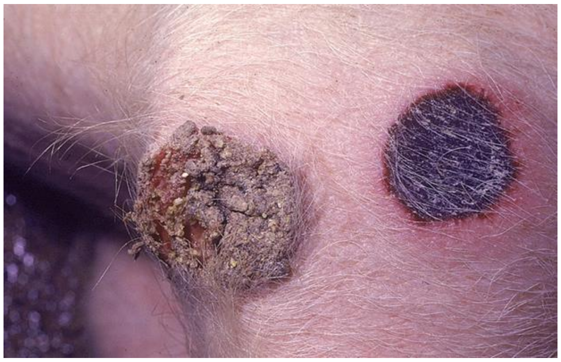

Figure 9b: Necrotic skin lesions.

Photo source: Plum Island Animal Disease center

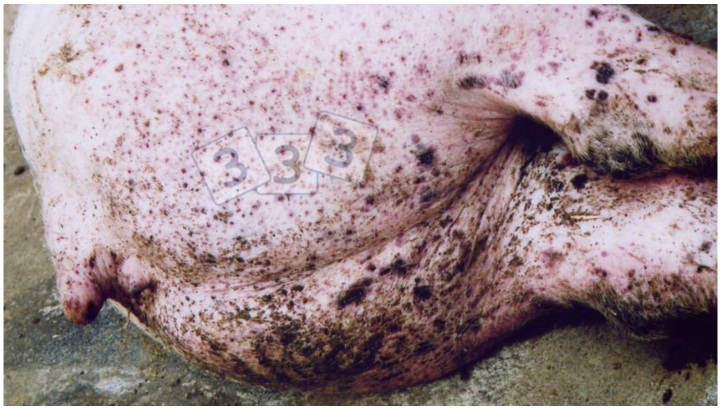

Figure 9c: Necrotizing dermatitis.

Photo source: https://pig333.com/pathology-atlas/

4. A detailed clinical scoring scheme to capture information collected at antemortem inspection will be provided.

Specific standard operating procedures for tissue collection

As a general principle, clean and thoroughly disinfect your sampling equipment, *e.g.,* knives, scalpel handles, scissors, and forceps, between animals, and all your personal protective gear, *e.g.,* overalls and gum boots, after your day's work. You will be provided with an appropriate disinfectant and more detail on how to use the disinfectant will be provided during the face-to-face training. The disinfectants that are known to inactivate ASFV and their contact times are

mentioned in **Table 1**. The contact time is the amount of time each disinfectant needs to be left on the equipment to ensure that all the microbes are inactivated[16]. After a thorough cleaning with soap and water followed by chemical disinfection of equipment, all items should be autoclaved to ensure sterilization. Also, change your gloves between animals sampled. This will prevent cross-contamination.

Table 1: Disinfectants that are able to kill African swine fever virus [17,18]

Active ingredient(s)(Concentration)	Trade name	Contact time
Sodium chloride (1.5%) & Potassium peroxymonosulfate (21.41%)	Virkon - S	10 minutes
Sodium dichloro-s-triazinetrione (48.21%)	Trade names may vary by manufacturer e.g., Klor-Kleen, Klorsept	30 minutes
Sodium hypochlorite (0.3%)	Trade names may vary by manufacturer e.g., JIK	15 minutes for non-porous objects/surfaces, and 30 minutes for porous objects/surfaces
Citric acid (3%)	Trade names may vary by manufacturer	15 minutes for non-porous objects/surface, and 30 minutes for porous objects/surfaces
Hydrogen peroxide (4.25%)	Trade names may vary by manufacturer	5 minutes
Thymol (0.05%)	Benefect Botanical Daily Cleaner Disinfectant Spray	15 minutes

i) Collecting the spleen and gastro-hepatic lymph node.

1. General principle: once the abdomen is opened, visually examine the abdominal viscera *in situ* (as they are/in their place) if possible.
2. Then, before handling any other organ/tissue, identify the spleen and the liver and locate the gastro-hepatic lymph node (between the stomach and the liver).

3. Afterwards, proceed to aseptically ("cleanly") remove approximately 5cm × 5cm piece of the spleen and the gastro-hepatic lymph node and place them into the tissue collection bags.

ii) Collecting the mandibular lymph nodes and the tonsils

1. When the head is separated from the rest of the animal, look for the submandibular and retropharyngeal lymph nodes (see the location of the two lymph nodes in **Figure 10**).
2. Make a cut along the medial aspect of both mandibles to free up the tongue.
3. Pull the tongue ventrally and caudally and then cut between the hyoid bones to disarticulate the hyoid apparatus.
4. Identify the tonsils, located on the dorsal aspect of the oral cavity, caudal to the hard palate. Remove the desired part of the tonsils and place it in a well labeled collection bag and place in the cool box. See **Figure 11a-c** for more guidance on how to remove the tonsils.
5. If you see a lesion, take as much as you can alongside some healthy tissue. If there is no lesion observed, take a piece of healthy tissue.

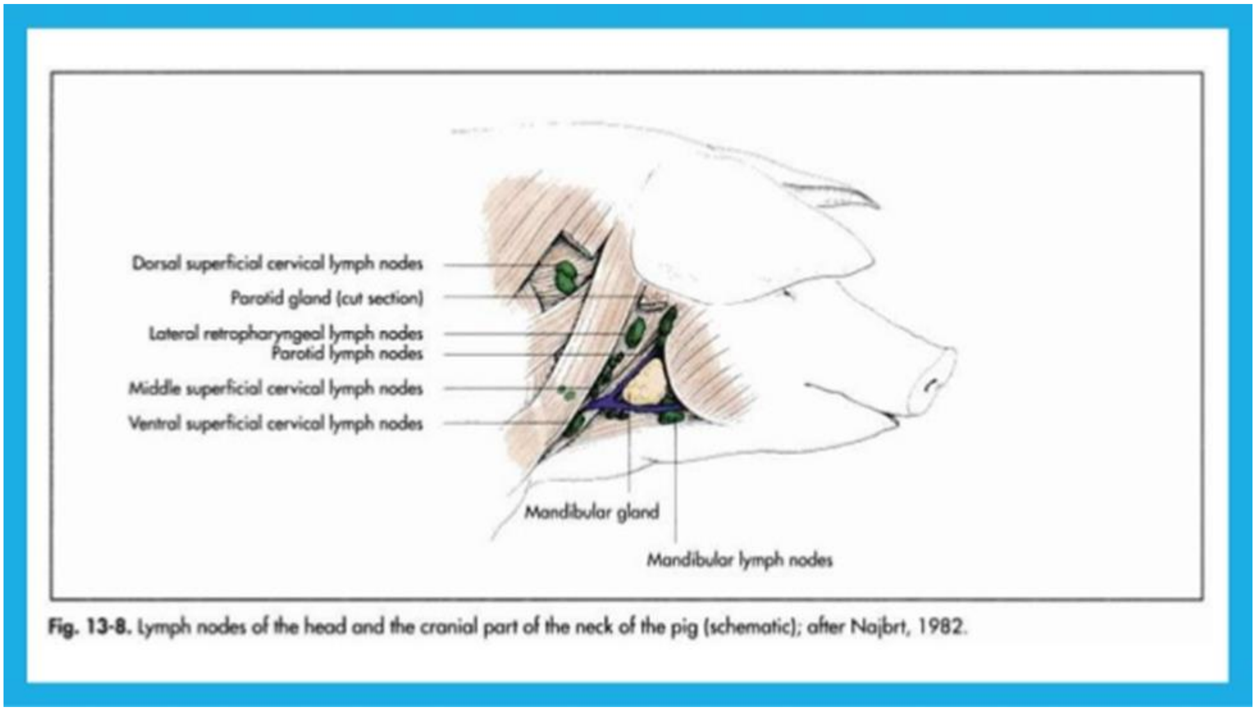

Figure 10. Location of lymph nodes of the neck and head.

Photo credit: Najbrt (1982) as adapted in Hamza (2018). https://www.slideshare.net/LuayHammza/the-lymphatic-system-89962537.

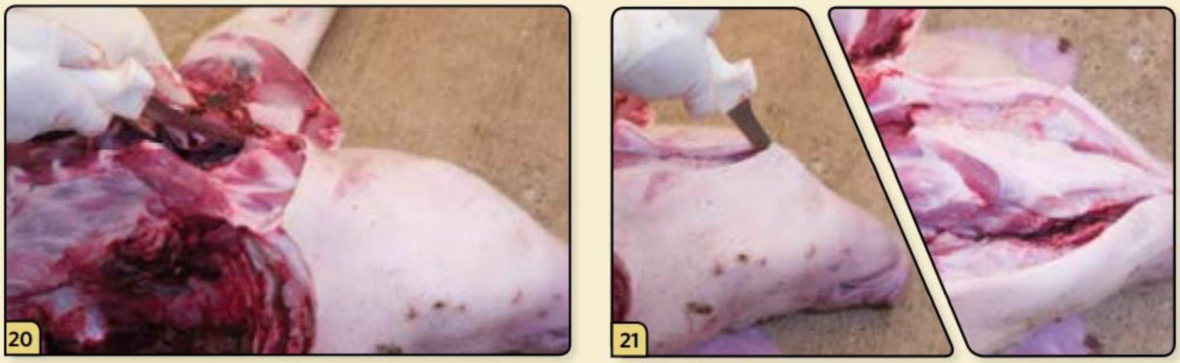

Extend the cut up to the level of the mandibles.

Cut along the medial aspect of both mandibles to free up the tongue.

Figure 11a: Initial cut up from the neck into the mandible to collect the lymph nodes of the head.

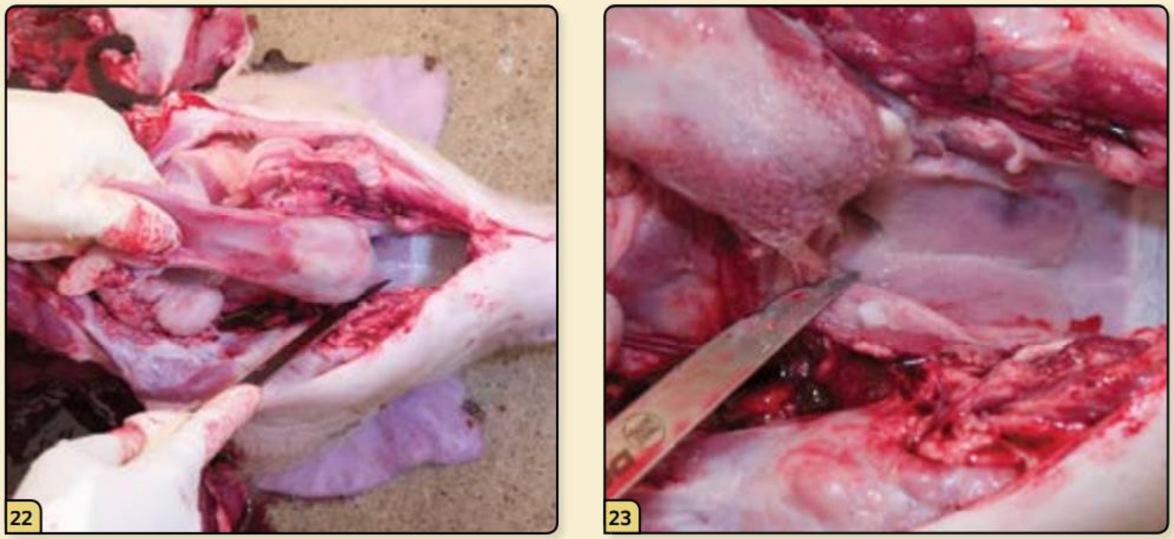

Pull the tongue ventrally and caudally to expose the oral cavity for inspection.

Cut between the hyoid bones to disarticulate the hyoid apparatus.

Figure 11b: How to expose the tonsils from a ventral position during a necropsy of swine

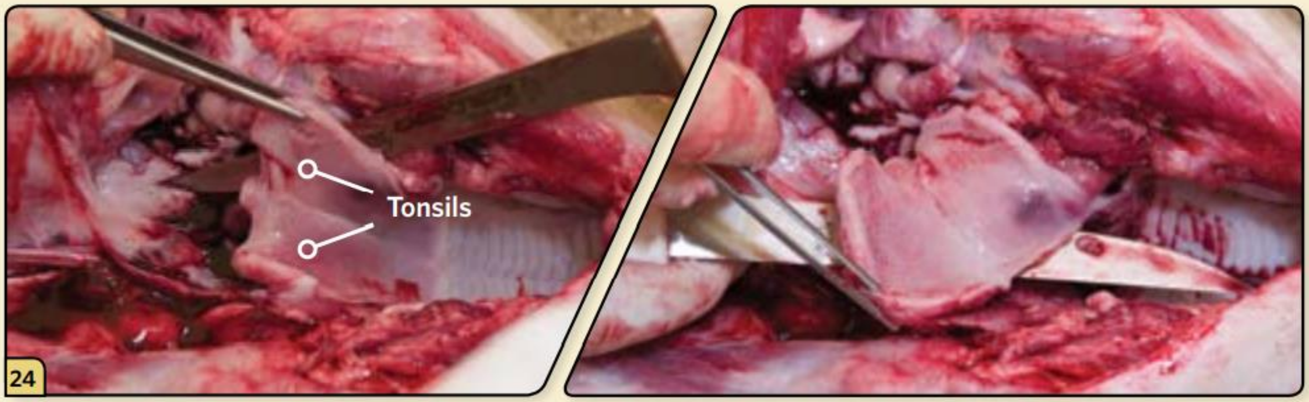

Identify the tonsils, located on the dorsal aspect of the oral cavity, caudal to the hard palate. Remove the tonsils and submit them for microbiology and histopathology.

Figure 11c: Removal of the tonsil of swine

Figures 11a-c: Extracted from the US Department of Agriculture, Foreign Animal Disease Investigation Manual, 2014: https://www.usdatraining.com/powerpoint_docs/FADD_Manual_digital.pdf.

Postmortem inspection

1. Ensure that the postmortem examination is carried out in a well-lit area. It is likely that you will just be looking at the organ pluck and the hanging carcass.
2. During the postmortem inspection, you should look out for the lesions shown in the images provided below. The common lesions you should look out for include:

a) Hemorrhage of the gastro-hepatic lymph node. See arrow in the picture below.

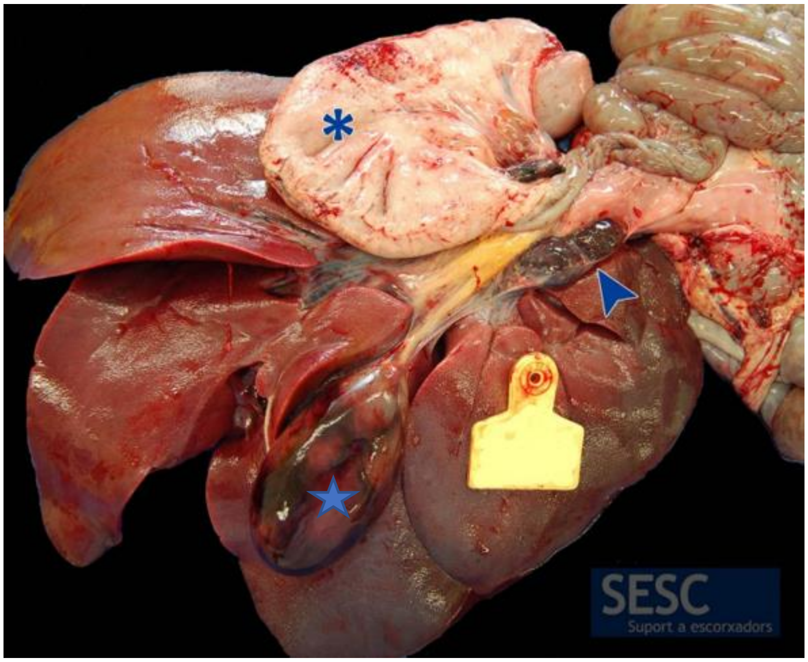

Figure 12a: The blue arrow shows hemorrhage of the gastro-hepatic lymph node. You can also see hemorrhage of the gallbladder wall as well as petechiae in the stomach wall (blue asterisk).

Picture source: http://www.cresa.cat/blogs/sesc/lesions-de-pesta-porquina-africana/?lang=en

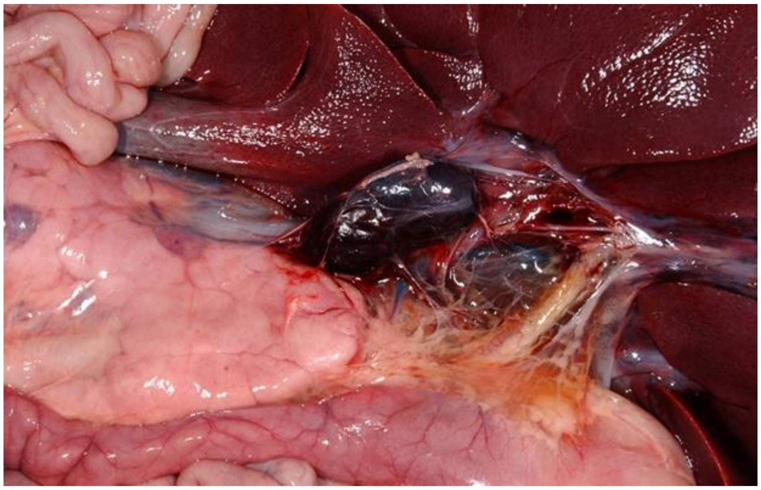

Figure 12b. Hemorrhagic gastro-hepatic lymph node.

Photo source: Plum Island Animal Disease Center.

b) Hemorrhagic splenomegaly. This is most characteristic of acute ASF. See pictures below.

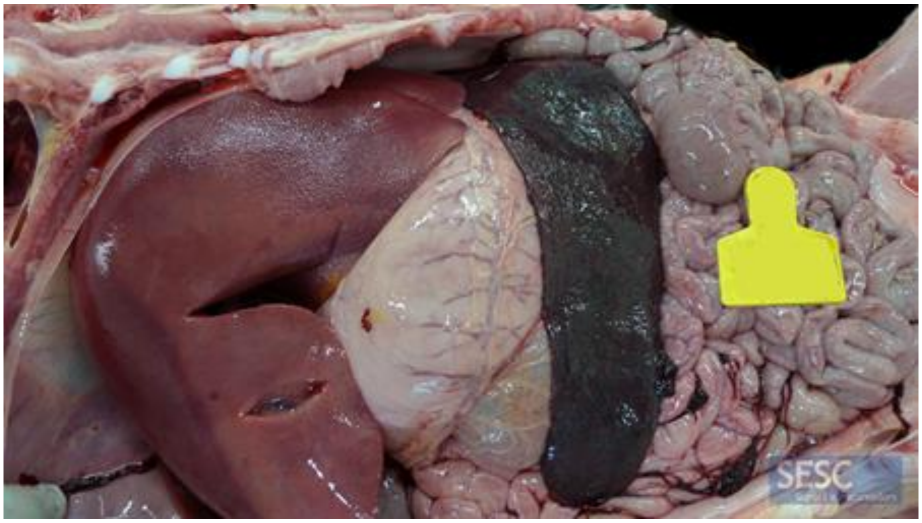

Figure 13a: Hemorrhagic splenomegaly in ASF.

Photo source: http://www.cresa.cat/blogs/sesc/lesions-de-pesta-porquina-africana/?lang=en

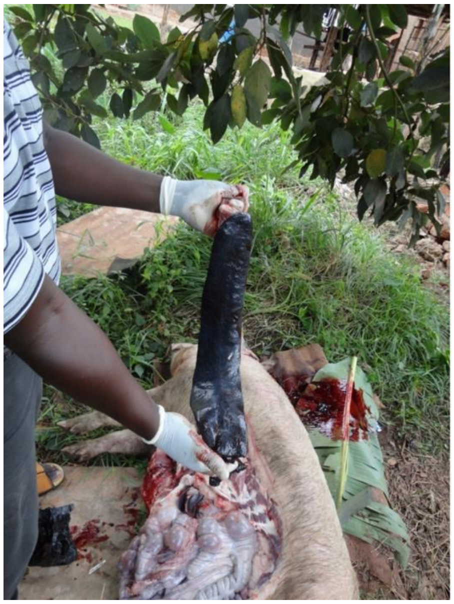

Figure 13b. Markedly enlarged and hemorrhagic spleen in a pig in Kanyanya, peri-urban Kampala.

Photo source: Ekakoro, 2013

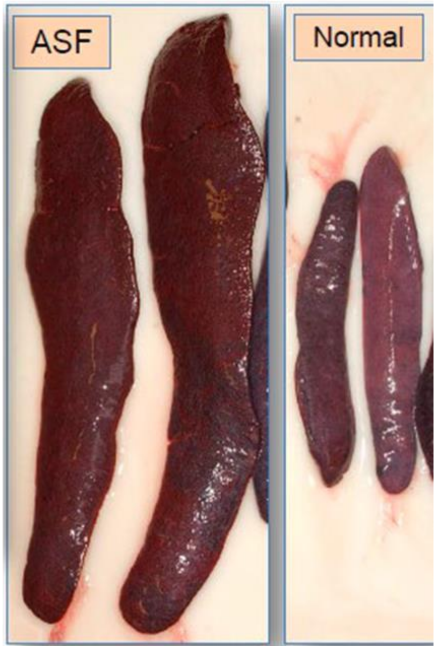

Figure 13c. Markedly enlarged and hemorrhagic spleen of ASF compared with normal spleens.

Photo source: Plum Island Animal Disease Center

c) Pulmonary edema as shown in the pictures below.

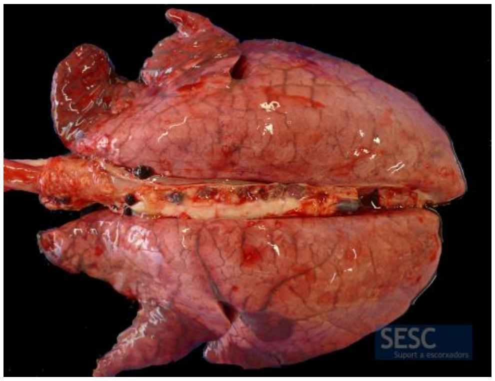

Figure 14a: This picture shows lungs that have not collapse. This is evidence of intense pulmonary edema. You can see evidence of interstitial edema between lung lobules.

Photo source:
http://www.cresa.cat/blogs/sesc/lesions-de-pesta-porquina-africana/?lang=en

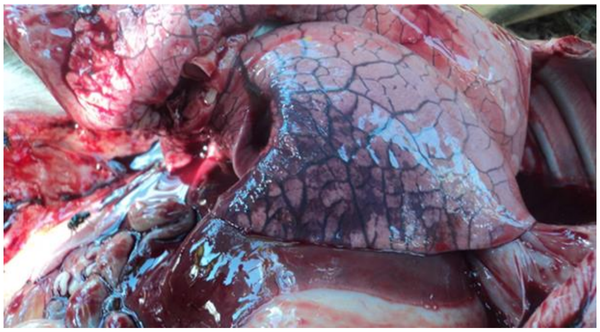

Figure 14b: This lung has a shiny appearance, with marked interlobular divisions. Sections of the lung is congested.

Photo Source: Ekakoro, 2013: Field postmortem examination of a pig during an ASF outbreak in Kanyanya, Peri-urban Kampala.

d) Perirenal edema is observed as shown in the picture below.

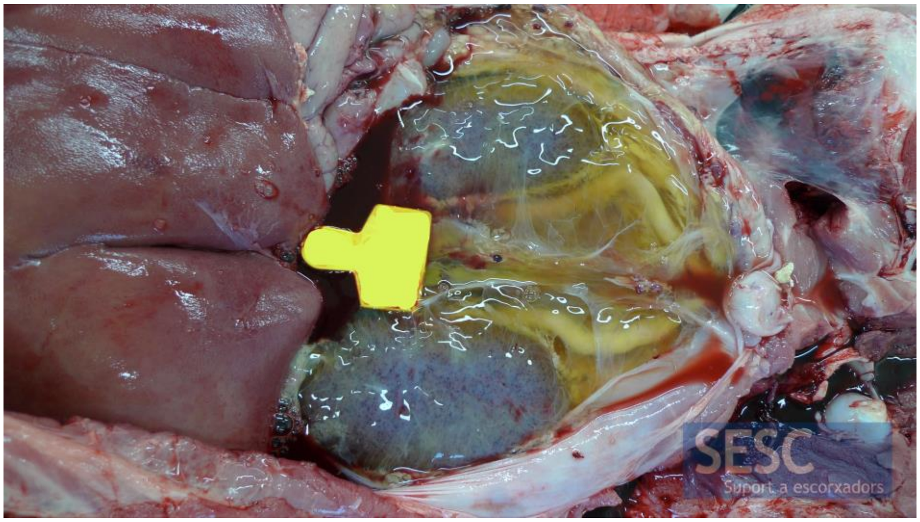

Figure 15: Peri-renal edema and multiple petechial hemorrhages in the kidneys.

Photo source: http://www.cresa.cat/blogs/sesc/lesions-de-pesta-porquina-africana/?lang=en

e) Hemorrhages of the renal lymph nodes as shown below.

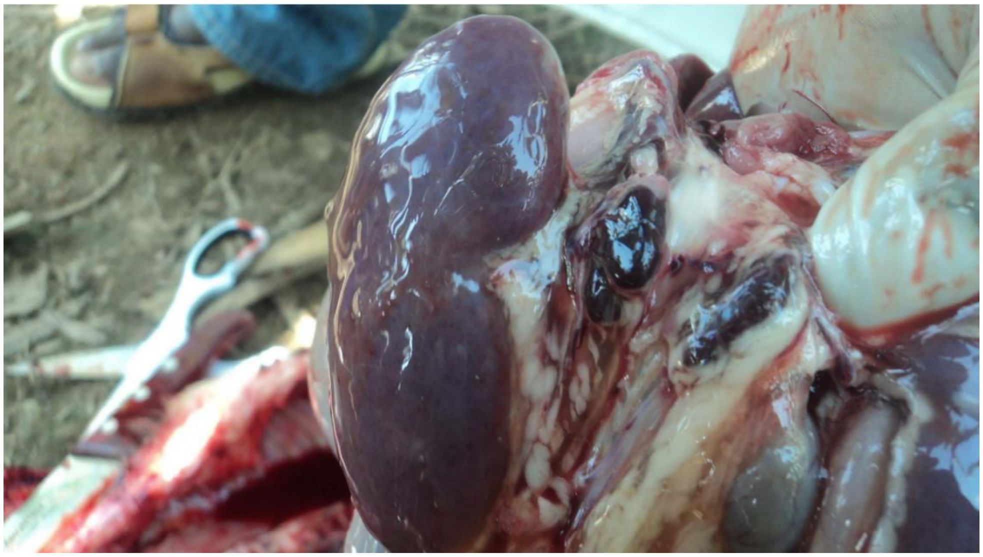

Figure 16. Hemorrhagic renal lymph nodes.

Photo: Ekakoro, 2013. Field postmortem examination of a pig during an ASF outbreak in Kanyanya, Peri-urban Kampala.

f) **Petechiation of the kidneys** as shown below.

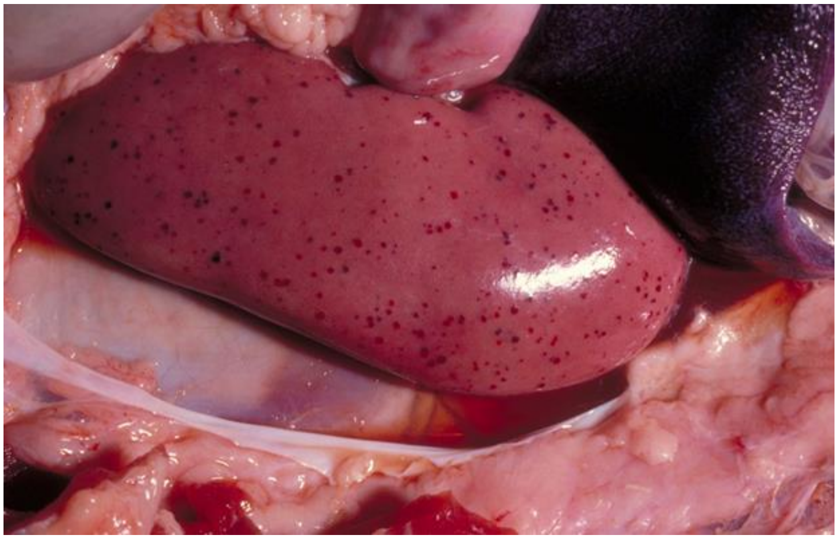

Figure 17. Pin-point hemorrhages in the kidneys.

Photo source: Plum Island Animal Disease Center.

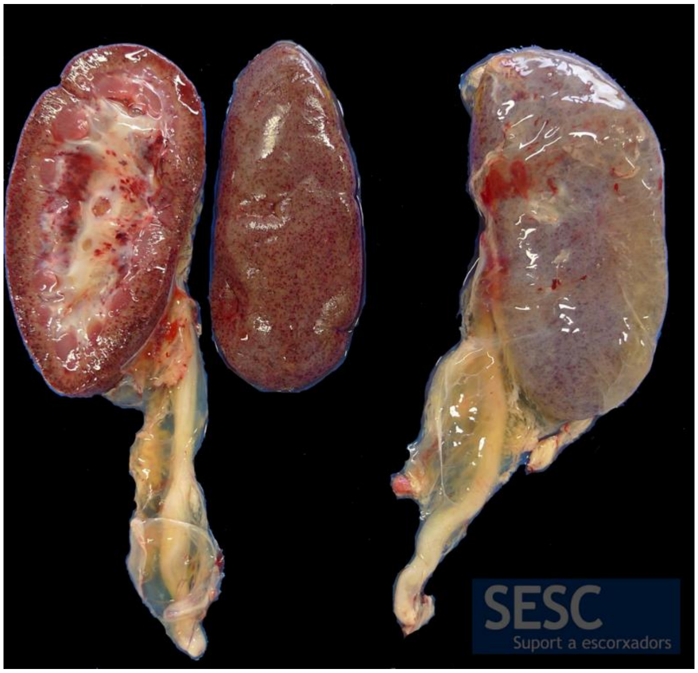

Figure 18: Petechiation in the kidney cortex and renal pelvis.

Photo source: http://www.cresa.cat/blogs/sesc/lesions-de-pesta-porquina-africana/?lang=en.

g) Hemorrhagic mesenteric lymph nodes and **hemorrhages** in the intestines as shown below.

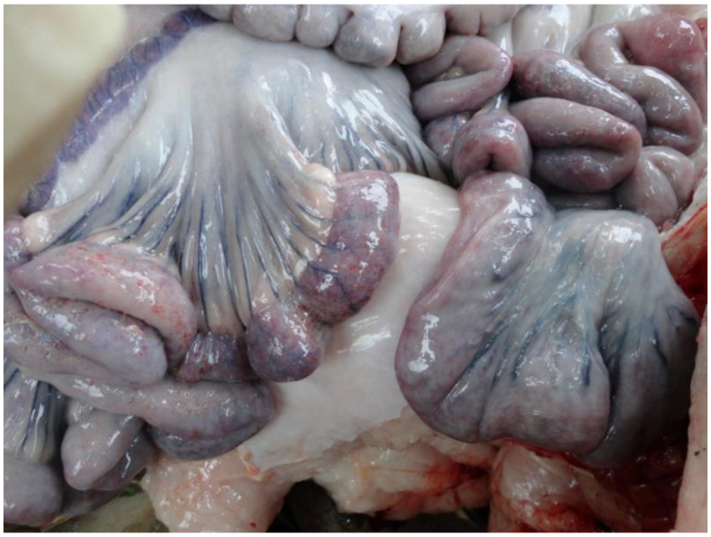

Figure 19: Hemorrhagic mesenteric lymph nodes and widespread petechial hemorrhages in the intestines.

Photo by Ekakoro, 2013. Field postmortem examination of a pig during an ASF outbreak in Kanyanya, Peri-urban Kampala.

h) Ascites shown below. (This may not be appreciable since the carcass will be hanging when opened.)

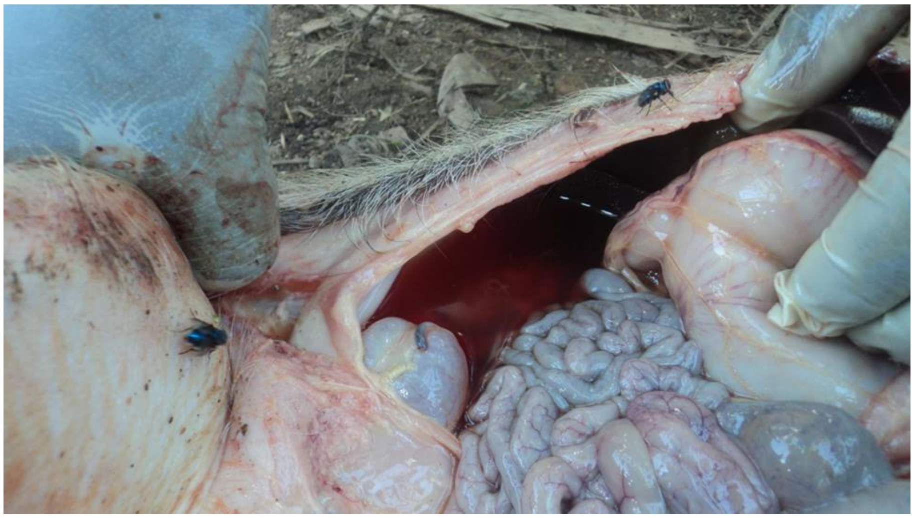

Figure 20: Ascites.

Photo by Ekakoro, 2013. Field postmortem examination of a pig during an ASF outbreak in Kanyanya, Peri-urban Kampala.

i) Hydropericardium and hemorrhages on the pericardium and heart

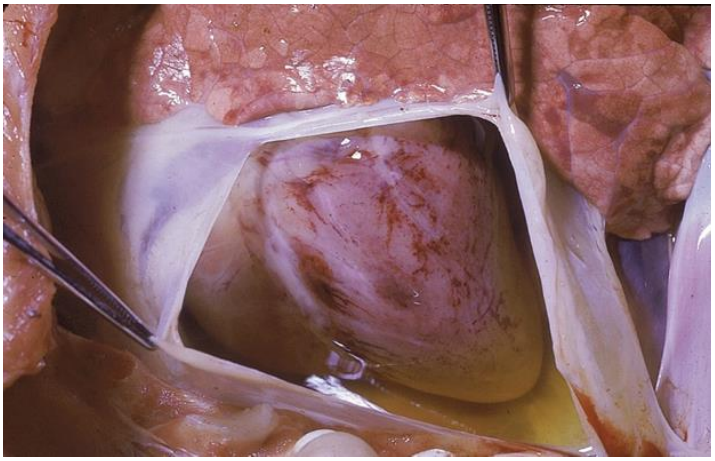

Figure 21: Straw-colored fluid in the pericardial cavity and hemorrhages in the epicardium.

Photo source: Plum Island Animal Disease Center.

j) **Edema of the gallbladder wall and surrounding connective tissue**

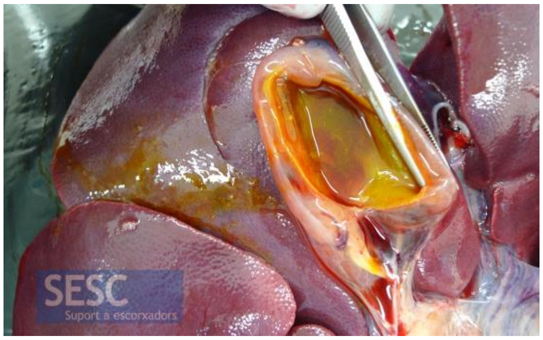

Figure 22a: Edema of the gall bladder wall.

Photo source: http://www.cresa.cat/blogs/sesc/lesions-de-pesta-porquina-africana/?lang=en.

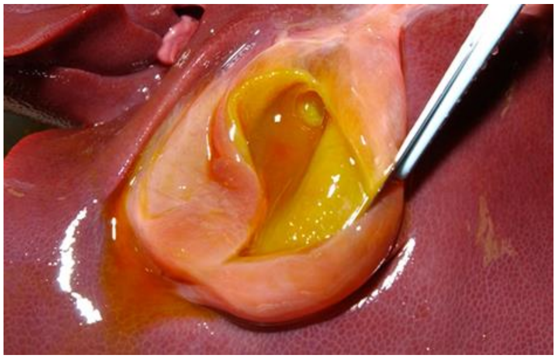

Figure 22b: Edema of the gall bladder wall.

Photo source: Plum Island Animal Disease Center.

k) Hemorrhages in the mandibular lymph nodes

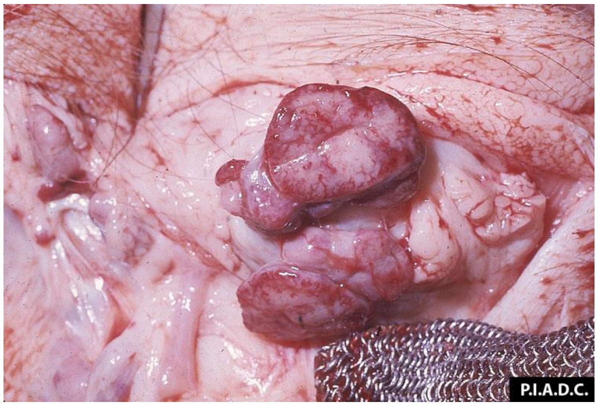

Figure 23: Moderate peripheral hemorrhages in mandibular lymph nodes.

Photo source: Iowa University, CFSPH.

l) Necrosis of the tonsils.

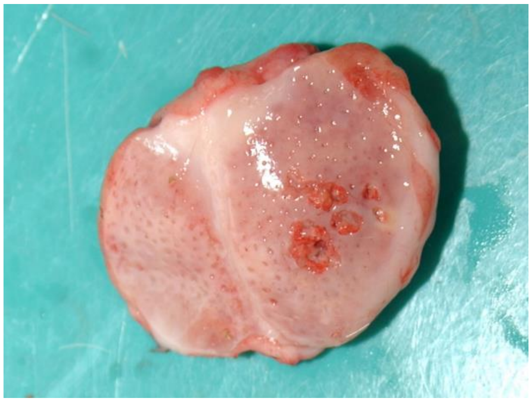

Figure 24a: Necrotic lesions on the tonsil.

Photo Source: Plum Island Animal Disease Center.

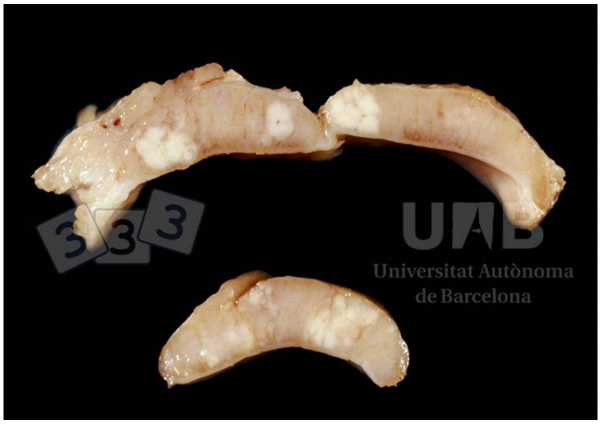

Figure 24b: Shows necrotic tonsilitis in ASF.

Photo source: [*https://pig333.com/pathology-atlas/*](https://pig333.com/pathology-atlas/)

Data collection tools

You will be provided with data collection tools (**see appendix**) consisting of the following items.

a) Pig biodata.

b) Clinical scoring.

c) Gross pathological scoring.

Funding

This project is funded by the Defense Threat Reduction Agency (DTRA) of the United States Department of Defense.

Acknowledgements

We would like to thank all the clinicians and pathologists in Uganda and the United States who reviewed this training manual.

Photo Sources

- Veterian Key: https://veteriankey.com/porcine-clinical-procedures/
- Plum Island Animal Disease center (PIADC): https://www.dhs.gov/science-and-technology/plum-island-animal-disease-center
- The Center for Food Security and Public Health (CFSPH): http://www.cfsph.iastate.edu/
- Pig333.com: https://www.pig333.com/
- http://www.cresa.cat/blogs/sesc/lesions-de-pesta-porquina-africana/?lang=en

References

1. Access online: OIE - World Organisation for Animal Health. Accessed November 11, 2020. https://www.oie.int/standard-setting/terrestrial-manual/access-online/

2. Salguero FJ. Comparative Pathology and Pathogenesis of African Swine Fever Infection in Swine. *Front Vet Sci*. 2020;7:282. doi:10.3389/fvets.2020.00282

3. Burrage TG. African swine fever virus infection in Ornithodoros ticks. *Afr Swine Fever Virus*. 2013;173(1):131-139. doi:10.1016/j.virusres.2012.10.010

4. Achenbach JE, Gallardo C, Nieto-Pelegrín E, et al. Identification of a New Genotype of African Swine Fever Virus in Domestic Pigs from Ethiopia. *Transbound Emerg Dis*. 2017;64(5):1393-1404. doi:10.1111/tbed.12511

5. Quembo CJ, Jori F, Vosloo W, Heath L. Genetic characterization of African swine fever virus isolates from soft ticks at the wildlife/domestic interface in Mozambique and identification of a novel genotype. *Transbound Emerg Dis*. 2018;65(2):420-431. doi:10.1111/tbed.12700

6. Norbert Mwiine F, Nkamwesiga J, Ndekezi C, Ochwo S. Molecular Characterization of African Swine Fever Viruses from Outbreaks in Peri-Urban Kampala, Uganda. Brown JC, ed. *Adv Virol*. 2019;2019:1463245. doi:10.1155/2019/1463245

7. Costard S, Mur L, Lubroth J, Sanchez-Vizcaino JM, Pfeiffer DU. Epidemiology of African swine fever virus. *Afr Swine Fever Virus*. 2013;173(1):191-197. doi:10.1016/j.virusres.2012.10.030

8. Constable PD, Hinchcliff KW, Done SH, Grünberg W, eds. 21 - Systemic and Multi-Organ Diseases. In: *Veterinary Medicine (Eleventh Edition)*. W.B. Saunders; 2017:2002-2214. doi:10.1016/B978-0-7020-5246-0.00021-8

9. African Swine Fever - Generalized Conditions. Merck Veterinary Manual. Accessed November 11, 2020. https://www.merckvetmanual.com/generalized-conditions/african-swine-fever/african-swine-fever?query=african%20swine%20fever

10. Sánchez-Vizcaíno JM, Laddomada A, Arias ML. African Swine Fever Virus. Wiley Online Library. Published March 29, 2019. Accessed November 11, 2020. https://onlinelibrary-wiley-com.proxy.library.cornell.edu/doi/abs/10.1002/9781119350927.ch25

11. Sánchez-Vizcaíno JM, Mur L, Gomez-Villamandos JC, Carrasco L. An Update on the Epidemiology and Pathology of African Swine Fever. *J Comp Pathol*. 2015;152(1):9-21. doi:10.1016/j.jcpa.2014.09.003

12. Binns C, Low WY. Public Health Implications of African Swine Fever in Asia. *Asia Pac J Public Health*. 2019;31(8):677-678. doi:10.1177/1010539519889539

13. Bellini S, Rutili D, Guberti V. Preventive measures aimed at minimizing the risk of African swine fever virus spread in pig farming systems. *Acta Vet Scand*. 2016;58(1):82. doi:10.1186/s13028-016-0264-x

14. FADD_Manual_digital.pdf. Accessed November 23, 2020. https://www.usdatraining.com/powerpoint_docs/FADD_Manual_digital.pdf

15. Acosta K. sop-swine-blood-collection. https://ouv.vt.edu/content/dam/ouv_vt_edu/sops/large-animal/sop-swine-blood-collection.pdf. :6.

16. The importance of contact time and visible wetness to ensure effective disinfection. Accessed December 2, 2020. https://www.beckershospitalreview.com/quality/the-importance-of-contact-time-and-visible-wetness-to-ensure-effective-disinfection.html

17. De Lorenzi G, Borella L, Alborali GL, Prodanov-Radulović J, Štukelj M, Bellini S. African swine fever: A review of cleaning and disinfection procedures in commercial pig holdings. *Res Vet Sci*. 2020;132:262-267. doi:10.1016/j.rvsc.2020.06.009

18. Birnbaum NG-A. Disinfectants Approved For Use Against African Swine Fever Virus In Farm Settings. Published online 2020:3.

Appendix: Abattoir Data collection form

An evaluation of African swine fever presentation and distribution in Uganda: Abattoir Data collection form

PI: Dr. Karyn Havas, kah47@cornell.edu
Co-PI: Dr. Eddie Wampande, ewampande@yahoo.co.uk

Name of abattoir………………………………………………………………………………….........................

GPS coordinates: Latitude…………………………………………….Longitude……….................................

Name of sample collector…………………………………………………………………………

A) Pig biodata

1. Sampling date (day/month/year)

   …………………………………………………………………………………………………

2. Sampling time (e.g., 8:30am)

   ………………………………………………………………………….................................

3. Environmental temperature at the abattoir at the time of sampling (Celsius)……………..

4. Ear tag ID (Should be identical to sample ID (whole blood, serum, organ samples (e.g., lymph nodes)

   ………………………………………………………………………...........................

5. Pig breed: Local ☐

   Exotic ☐

   Cross (mixed) ☐

   Unknown ☐

6. Color of the pig: All black ☐

Black and white ☐

All white ☐

Other color ☐ Specify [ ]

---

7. Sex: Male……………… Castrate (Yes/No)………………………………………………

Female……………….

---

8. The origin source of the pig:

Village……

Subcounty……

District……..

Region (e.g., central)……………………………………………………………………………………………

---

9. Where was the pig bought (sourced)?

Directly from a smallholder producer (1-3 pigs) ☐

Directly from a medium-scale producer (4-11 pigs) ☐

Directly from a large-scale producer (> 11 pigs) ☐

From a livestock market ☐ Mention location [ ]

Other (specify) [ ]

---

10. What was the pre-purchase health status of the pig?

Apparently healthy ☐

Sick ☐

If sick, what sign(s) did you observe?...

……..

---

11. What was the health status of the pig since the purchase?

Apparently healthy ☐

Sick ☐

If sick, what sign(s) did you observe?...

……..

---

12. Duration of ownership of the pig by the farmer (if known, as self-reported by the farmer), otherwise leave blank.

Duration…………………Units (circle one of the following units: days, weeks, months, years)

13. Age of the pig (as reported by the farmer), otherwise leave blank.

Age……………………Units (circle one of the following units: days, weeks, months, years)

B) Clinical scoring

Clinical signs/antemortem picture	Description/comments	Score
Depression/Liveliness	Pig appears normal with no evidence of depression.	0
	The pig is quiet and less responsive to its environment	1
	The pig is moribund.	2
	Any other distinct observation(s) (specify).	
Walking	Well-coordinated movements	0
	Hesitant to walk.	1
	Distinct staggering gait/hind lameness, but able to walk.	2
	Massive lameness, unable to walk.	3
	Any other distinct observation(s) (specify).	
Diarrhea	No diarrhea.	0
	Non-bloody diarrhea/evidence of non-bloody diarrhea.	1
	Bloody diarrhea/evidence of bloody diarrhea.	2
	Any other distinct observation(s) (specify).	
Vomiting	Not vomiting/no evidence of vomiting.	0
	Vomiting/there is evidence of vomiting.	1
	Bloody vomiting	2
Fever (ascertained via rectal temperature)<br>Enter the rectal temperature in the space below.<br><br>……………………………………	Temperature is below 38.7C	4
	38.7-39.8C (normal/no fever).	0
	39.9-40.5C (mild).	1
	>40.5-41C (moderate).	2
	>41C (Severe).	3
Body condition	Normal or over conditioned (fat).	0
	Thin. Can see depressions between the hip and vertebrae but requires pressure to feel bones on palpitation.	1
	Emaciated. Hip and vertebral bones are prominent and easy to feel on palpation.	2
Joints	Joint(s) normal.	0

	Joint(s) swollen.	1
	Severe joint swelling and lameness.	2
	Which joint(s) is swollen? Circle/mark all that apply on the picture provided (**Figure 1**).	
Breathing	Breathing pattern is normal.	0
	Labored breathing.	1
	Agonal/gasping breathing	2
	Any other observation(s) (specify).	
Cough	Not coughing.	0
	Coughing.	1
	Any other observation(s) (specify).	
Nasal discharges	No nasal discharges.	0
	Serous or seromucous nasal secretions.	1
	Blood-tinged nasal secretions	2
	Any other observations (specify).	
*Cyanosis/hyperemia/hemorrhages of the skin (particularly of the ears, nose, abdomen, flanks, legs, and tail).	No signs of hyperemia	0
	Reddened or purplish skin areas around the edges of the ears.	1
	Purplish-colored skin discoloration observed, around the abdomen, flanks, and ears.	2
	Dark purple to black-red discoloration of the skin across the abdomen, sides, and flanks.	3
	Any other distinct observation(s) (specify).	
Skin necrosis	No signs of skin necrosis.	0
	Raised reddened circular lesions.	1
	Necrotic skin circular lesions.	2
	Any other distinct observation(s) (specify).	
Eye/conjunctiva	Light pink.	0
	Reddened, clear secretion.	1
	Highly inflamed, turbid secretion.	2
	Highly inflamed, purulent secretion, prominent blood vessels.	3
	Any other distinct observation(s) (specify).	

*Cyanosis/hyperemia/hemorrhages may not be clearly observable in some black colored pigs.

Clinical signs scoring scheme modified from McCleary 2020, Howey et al, 2013; Galindo-Cardiel et al, 2013; Mittelholzer and Moser, 2000. ASF body condition scoring as described by Olesen et al, 2017.

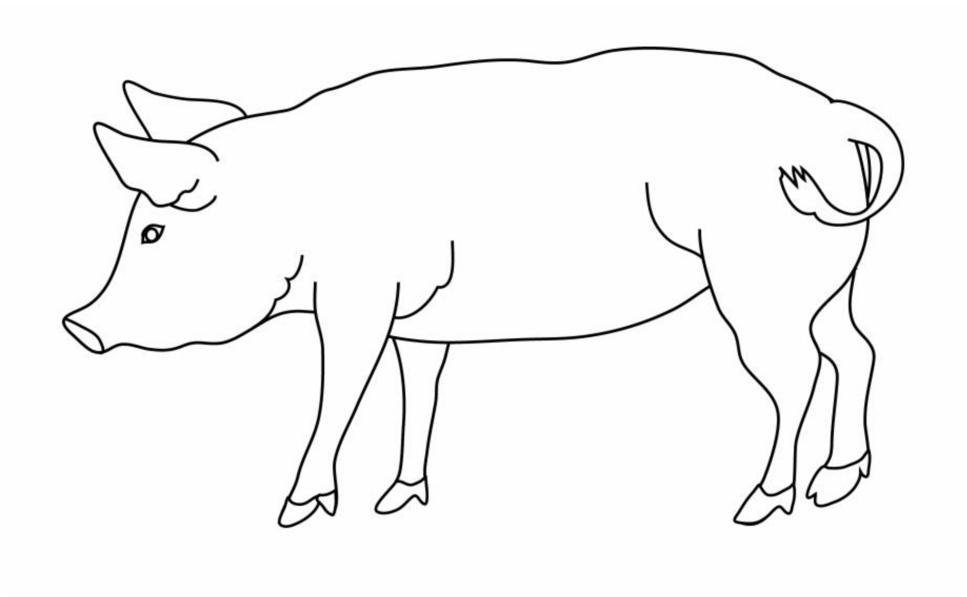

http://clipart-library.com/clip-art/20-205689_pig-outline-png-transparent-background-pigs-drawing-black.htm

Figure 1: Image to be used to mark affected joints with an X.

Credit: Clipart-library.com

C) Gross pathological scoring of ASFV lesions.

Organ/tissue	Gross pathological observation	Description	Category (Score)
Lungs	Lack of collapse	No rib impressions.	0
		Mild rib impressions.	1
		Clear rib impressions.	2
	Edema	No apparent lung edema or froth in trachea.	0
		The lung has a moist and shiny appearance.<br><br>Some froth in the trachea.<br><br>Interlobular divisions are evident.	1

		The lungs are heavy. A lot of froth in the trachea. Interlobular divisions are marked.	2
		Other distinct observations(s) (specify).	
	Hemorrhage	No hemorrhages observed.	0
		Patches of reddened tissue seen throughout the lungs.	1
		Widespread reddened section of lung with evidence of hemorrhage.	2
		Other distinct observations(s) (specify).	
	Cranio-ventral Consolidation	No consolidation.	0
		Mild consolidation in one/both lungs.	1
		Severe consolidation in one/both lungs.	2
Kidneys	Hemorrhages	No hemorrhages on kidney surface.	0
		Some pinpoint (petechial) hemorrhages evident on kidney surface.	1
		Petechiation that is widespread on the surface of the kidney.	2
		Other distinct observation(s) (specify).	
Spleen	Enlargement	Spleen is of normal size.	0
		Mild to moderately enlarged spleen. Splenic capsule shows some cracking when spleen bent.	1
		Spleen is markedly enlarged (gigantic). Spleen breaks easily when bent.	2
		Other distinct observation(s) (specify).	
	Hemorrhagic	No observable changes in spleen color.	0

		Multiple areas (patches) of the spleen are dark colored, other parts are normal.	1
		The entire spleen is very dark red (almost black).	2
		Other distinct observation(s) (specify e.g., splenic fibrosis observed).	
Pericardial cavity	Hydropericardium	No fluid in the pericardial cavity.	0
		Straw-colored fluid in pericardial cavity.	1
		Blood-stained fluid in pericardial cavity.	2
		Not observable.	
		Other distinct observation(s) (specify).	
	Fibrinous pericarditis	No fibrinous (yellowish) exudate.	0
		Fibrinous (yellowish) exudate observed.	1
		Other distinct observation(s) specify.	
Hepato-gastric lymph node	Hemorrhage/edema	No hemorrhage/edema.	0
		Enlarged and edematous.	1
		Multifocal hemorrhages with a marbled appearance.	2
		Enlarged lymph node with diffused hemorrhage.	3
		Other distinct observation(s) (specify).	
Renal lymph nodes	Hemorrhage/edema	No hemorrhage/edema	0
		Enlarged and edematous	1
		Multifocal hemorrhages with a marbled appearance.	2
		Enlarged lymph node with diffused hemorrhage.	3
		Other distinct observation(s) (specify).	
Submandibular lymph nodes	Hemorrhages/edema	No hemorrhage/edema.	0
		Enlarged and edematous.	1
		Multifocal hemorrhages with a marbled appearance.	2

		Enlarged lymph nodes with diffused hemorrhage.	3
		Other distinct observation(s) (specify).	
Mesenteric lymph nodes	Hemorrhages/edema	No hemorrhage.	0
		Enlarged and edematous.	1
		Multifocal hemorrhages with a marbled appearance.	2
		Enlarged lymph nodes with diffused hemorrhage.	3
		Other distinct observation(s) (specify).	

Other postmortem observations

1. **Other organs with hemorrhages (excluding kidneys, lungs, spleen, lymph nodes).**

Hemorrhages observed in the specified organs: Select all that apply	Organ(s)	Description/observation	Score
	Intestinal serosa/outside surface of intestines	No hemorrhage.	0
		Hemorrhage present.	1
	Urinary bladder serosa/outside surface of the urinary bladder	No hemorrhage.	0
		Hemorrhage Present.	1
	Pericardium/epicardium	No hemorrhage.	0
		Hemorrhage present.	1
Other organs (exclude lung, kidneys, spleen, & lymph nodes)	List organ(s) below	Description/observation.	Score
		No hemorrhage.	0
		Hemorrhage present.	1

2. **Other organs/tissues where edema or necrosis is present.**

**Edema**	**Description/observation**	**Score**
Renal fat	No edema.	0
	Edema present.	1
	Not observable.	
Gall bladder	No edema.	0
	Edema present.	1
	Not observable.	
**Necrosis**	**Description/observation**	**Score**
Tongue	No evidence of necrosis.	0
	Multifocal and whitish areas observed (necrosis present).	1

Tonsils	No abnormality observed.	0
	Multifocal and whitish areas observed in tonsils (necrosis present).	1
	The tonsil is red/purple, and/or marked exudate is observed	2
	Not observable.	

Scoring scheme based on postmortem lesions as described by Salguero 2020, Guinat et al, 2014, Galindo-Cardiel et al, 2013, and Sánchez-Vizcaíno and Neira 2012.
